# Position Statement on Indications of Echocardiography in Adults - 2019

**DOI:** 10.5935/abc.20190129

**Published:** 2019-07

**Authors:** Silvio Henrique Barberato, Minna Moreira Dias Romano, Adenalva Lima de Souza Beck, Ana Clara Tude Rodrigues, André Luiz Cerqueira de Almeida, Bruna Morhy Borges Leal Assunção, Eliza de Almeida Gripp, Fabio Villaça Guimarães Filho, Henry Abensur, José Maria Del Castillo, Marcelo Haertel Miglioranza, Marcelo Luiz Campos Vieira, Márcio Vinicius Lins de Barros, Maria do Carmo Pereira Nunes, Maria Estefania Bosco Otto, Renato de Aguiar Hortegal, Rodrigo Bellio de Mattos Barretto, Thais Harada Campos, Vicente Nicoliello de Siqueira, Samira Saady Morhy

**Affiliations:** 1 CardioEco-Centro de Diagnóstico Cardiovascular, Curitiba, PR - Brazil; 2 Quanta Diagnóstico e Terapia, Curitiba, PR - Brazil; 3 Faculdade de Medicina de Ribeirão Preto da Universidade de São Paulo (FMRP-USP), Ribeirão Preto, SP - Brazil; 4 Instituto de Cardiologia do Distrito Federal, Brasília, DF - Brazil; 5 Fundação Universitária de Cardiologia (ICDF/FUC), Brasília, DF - Brazil; 6 Hospital das Clínicas da Faculdade de Medicina da Universidade de São Paulo (HC-FMUSP), São Paulo, SP - Brazil; 7 Santa Casa de Misericórdia de Feira de Santana, Feira de Santana, BA - Brazil; 8 Instituto do Câncer do Estado de São Paulo (ICESP), São Paulo, SP - Brazil; 9 Hospital Sírio-Libanês, São Paulo, SP - Brazil; 10 Hospital Pró-Cardíaco, Rio de Janeiro, RJ - Brazil; 11 Hospital Universitário Antônio Pedro, Niterói, RJ - Brazil; 12 DASA, São Paulo, SP - Brazil; 13 Faculdade de Medicina de Marília, Marília, SP - Brazil; 14 Hospital Beneficência Portuguesa de São Paulo, São Paulo, SP - Brazil; 15 Pronto Socorro Cardiológico de Pernambuco (PROCAPE - UPE), Recife, PE - Brazil; 16 Instituto de Cardiologia de Porto Alegre, Porto Alegre, RS - Brazil; 17 Instituto do Coração do Hospital das Clínicas da Faculdade de Medicina da Universidade de São Paulo (InCor-HCFMUSP), São Paulo, SP - Brazil; 18 Hospital Israelita Albert Einstein, São Paulo, SP - Brazil; 19 Faculdade de Saúde e Ecologia Humana (FASEH), Vespasiano, MG - Brazil; 20 Rede Materdei de Saúde, Belo Horizonte, MG - Brazil; 21 Hospital Vera Cruz, Belo Horizonte, MG - Brazil; 22 Universidade Federal de Minas Gerais, Belo Horizonte, MG - Brazil; 23 Instituto Dante Pazzanese de Cardiologia, São Paulo, SP - Brazil; 24 Diagnoson-Fleury, Salvador, BA - Brazil; 25 Hospital Ana Nery, Salvador, BA - Brazil; 26 Universidade Federal de São Paulo (UNIFESP), São Paulo, SP - Brazil

**Table:** 

Declaration of potential conflict of interest of authors/collaborators of Updated Geriatric Cardiology Guidelines of the Brazilian Society of Cardiology - 2019 If the last three years the author/developer of the Updated:							
Names Members of the Statement	Participated in clinical studies and/or experimental trials supported by pharmaceutical or equipment related to the guideline in question	Has spoken at events or activities sponsored by industry related to the guideline in question	It was (is) advisory board member or director of a pharmaceutical or equipment	Committees participated in completion of research sponsored by industry	Personal or institutional aid received from industry	Produced scientific papers in journals sponsored by industry	It shares the industry
Adenalva Lima de Souza Beck	No	No	No	No	No	No	No
Ana Clara Tude Rodrigues	No	No	No	No	No	No	No
André Luiz Cerqueira de Almeida	No	No	No	No	No	No	No
Bruna Morhy Borges Leal Assunção	No	No	No	No	No	No	No
Eliza de Almeida Gripp	No	No	No	No	No	No	No
Fabio Villaça Guimarães Filho	No	No	No	No	No	No	No
Henry Abensur	No	No	No	No	No	No	No
José Maria Del Castillo	No	No	No	No	No	No	No
Marcelo Haertel Miglioranza	No	No	No	No	No	No	No
Marcelo Luiz Campos Vieira	No	No	No	No	No	No	No
Márcio Vinicius Lins de Barros	No	No	No	No	No	No	No
Maria do Carmo Pereira Nunes	No	No	No	No	No	No	No
Maria Estefânia Bosco Otto	No	No	No	No	No	No	No
Minna Moreira Dias Romano	No	No	No	No	No	No	No
Renato de Aguiar Hortegal	No	No	No	No	No	No	No
Rodrigo Bellio de Mattos Barretto	No	No	No	No	No	No	No
Samira Saady Morhy	No	No	No	No	No	No	No
Silvio Henrique Barberato	No	No	No	No	No	No	No
Thais Harada Campos	No	No	No	No	No	No	No
Vicente Nicoliello de Siqueira	No	No	No	No	No	No	No

## 1. Introduction

In accordance with the “Standards for the Elaboration of Guidelines, Positions and Normations” sanctioned by the Brazilian Society of Cardiology, this document was written to update the “Echocardiography Indication Guidelines” of 2009. The new document is not intended to be a comprehensive review of echocardiography, but rather an indispensable basic guide to support the rational clinical decision-making of the physician requesting the exam for adult patients. Although it considers the recent technological advances of echocardiography, its purpose is not to describe in detail echocardiography methods, but to summarize in a clear and concise way the main situations in which echocardiography brings benefit to the diagnosis and/or therapeutic orientation of the individual. This manuscript chose to highlight the class of recommendation, as described below:

- Class I: conditions for which there is conclusive evidence or, in the absence thereof, general agreement that the examination procedure is useful and safe.
- Class II: conditions for which there is conflicting evidence and/or divergence of opinion on the utility and/or safety of the examination.
- Class IIa: evidence or opinions favorable to the examination. Most experts approve.
- Class IIb: utility and/or safety less well established, with divergent opinions.
- Class III: conditions for which there is evidence or consensus that the examination is not useful and, in some cases, may even be harmful.

In addition, the level of evidence was also described, as follows:

- A: several concordant randomized clinical trials or robust meta-analyses;
- B: less robust meta-analysis data or single randomized clinical study or observational studies;
- C: expert opinion.

Thus, it was agreed that all the tables with recommendations for the use of echocardiography in the different clinical scenarios include columns with class of recommendation and level of evidence.

## 2. Evaluation of Heart Function and Structure

### 2.1. Left Ventricular Systolic Function

The analysis of left ventricular (LV) systolic function is a primary indication of the use of echocardiography. Echocardiographic analysis of LV systolic function can be performed using older techniques such as M-mode, 2D echocardiography, and even more modern techniques, such as three-dimensional (3D) echocardiography or research of myocardial deformation (strain). The M-mode has been used since the 1950s for cardiac structural analysis and provides widely standardized measurements^1,2^ of the dimensions of the cavity and the thickness of the LV. Thus, parameters of ventricular systole analysis are derived as: (1) percentage of systolic shortening of the left ventricular dimension, represented by the difference between the final diastolic and the final systolic dimensions, divided by the final diastolic one; (2) mean corrected circumferential shortening velocity, corresponding to the ratio between the percentage of systolic shortening of the left ventricular dimension divided by the ejection time corrected by the preceding R-R interval (ejection time divided by the square root of the R-R interval); (3) ventricular volumes at the end of systole and diastole, calculated from the method by Teichholz et al.;^3^ and (4) LV ejection fraction (LVEF), obtained from the difference between the diastolic and systolic ventricular volumes (volume ejected by systole), divided by the diastolic volume. The M-mode analysis is highly reproducible and presents high temporal resolution for the analysis of ventricles without spatial deformation.^4^ However, M-mode measurements, in general, adequately determine global systolic function only when there are no segmental changes, remodeling and/or geometric alterations of the LV.^4^ With the advent of 2D echocardiography, a greater amplitude of the LV spatial observation was acquired, allowing better analysis of the left ventricular systolic function, when compared to the one-dimensional analysis. This occurs in situations where there are changes in left ventricular geometry, such as apical aneurysm and other segmental changes resulting from coronary artery disease. The analysis of 2D left ventricular systolic function can be performed qualitatively (visual estimation) or quantitatively of LVEF. Visual estimation is highly dependent on the training of the operator, which can result in inaccuracy of reproduction of results. The quantitative method for volume and systolic function analysis of the most widespread and widely valid 2D-LV is the biplanar disc technique (modified Simpson’s rule), in which the total volume is calculated based on the sum of the volumes of small cylindrical discs in apical cuts 4 and 2 LV chambers, with the intention of minimizing the effects of modifying the ventricular geometry in LVEF calculation.^4^ Normal volume ​​and LVEF values, calculated using 2D echocardiography, show different values depending on gender. Thus, for men, the final LV diastolic volume is between 34 and 74 mL/m^2^, the final LV systolic volume is between 11 and 31 mL/m^2^ and LVEF is between 52 and 72%; LV final diastolic volume is between 29 and 61 mL/m^2^, LV final systolic volume is between 8 and 24 mL/m^2^ and LVEF is between 54 and 74%.^4^ The 2D LVEF analysis may present inaccuracies during the shortening of LV (foreshortening) or inadequate acoustic window, and when there are coexistent geometric changes in the apical 4 and 2 LV chambers.^4^ The analysis of LV segmental contractility by 2D echocardiography represents a semiquantitative technique to assess regional systolic function, which has shown good application in clinical practice, especially for stress echocardiography (calculus of parietal motility index, which integrates analysis of ventricular wall thickening and ventricular segment contractility). Tissue Doppler, a technique used in the analysis of diastolic function, can also be used to assess global and segmental LV systolic function. The systolic velocity of the ventricular myocardium (s-wave), when measured in the region of the mitral annulus, reflects the longitudinal myocardial systolic shortening and may be reduced early in patients with diastolic dysfunction and normal ejection fraction.^5^ This method may also be useful for ventricular synchrony analysis and as a complement in stress echocardiography, although it does not allow adequate evaluation of systolic function in the apical LV segments and depends on the angle of incidence of the ultrasound beam.

3D echocardiography represented an improvement over 2D echocardiographic observation of LV function, once it does not present the limitations of the 2D analysis in ventricles with altered geometry, in addition to being more reproducible and better correlated with the gold standard provided by nuclear magnetic resonance.^4,6-11^ Most recent algorithms allow the calculation of LVEF and LV volumes in a semi-automatic way, with great correspondence with the analysis performed by nuclear magnetic resonance.^12^ However, 3D echocardiography presents difficulties in relation to low temporal resolution and to the dependence on transthoracic echocardiographic image quality.^4^

The understanding and analysis of LV mechanics and systolic function can also be determined by the measurement of ventricular strain. Strain is defined as the modification of length of the myocardial segment (in %), considering the different spatial arrangements of the myocardial fibers. In this way, the longitudinal, circumferential and radial strain (for the respective longitudinal, circumferential and radial myocardial fibers) is calculated. The strain can be calculated for each of the LV segments or for all segments (global LV strain).^13,14^ The analysis of the cardiac mechanics can be performed from parameters derived from myocardial deformation, such as twist, torsion and LV rotation. In order to obtain ventricular strain, the most commonly used technique takes into account the movement of gray points in the myocardium during the cardiac cycle (speckle tracking technique).^13,14^ Global 2D strain has the advantages of not depending on the angle of the ultrasound beam (as in tissue Doppler) and of presenting an independent prognostic LVEF value.^4^ However, it does not yet present a standardized value of normality among the different manufacturers of echocardiography equipment.^4^ The LV global longitudinal strain (GLS) has been the one most commonly used in practice, which is useful for the detection of subclinical myocardial dysfunction, even when LVEF is preserved; for example: evaluation of cardiotoxicity after the use of chemotherapy for antineoplastic treatment, rejection after heart transplantation, severe aortic stenosis, hypertrophic cardiomyopathy and myocardium infiltrative diseases.^15-19^

### 2.2. Left Ventricular Diastolic Function

The evaluation of LV diastolic function is an integral part of routine echocardiography analysis, especially in patients with dyspnea or suspected heart insufficiency.^20,21^ Furthermore, in several cardiopathies, the diastolic dysfunction precedes the systolic one. Diastolic dysfunction is usually the result of altered relaxation, with or without reduction of restorative forces (early diastolic suction), and increased LV stiffness, leading to elevated LV filling pressures.^20^ When pulmonary capillary pressure exceeds 12 mmHg, or final diastolic LV pressure exceeds 16 mmHg, filling pressures are considered high.^21^ Elevation of filling pressures occurs as a compensatory response to maintain adequate cardiac output, and its estimation is important not only for the diagnosis of cardiac insufficiency but also for the definition of its severity and response to treatment.^21^ It is recommended that the non-invasive analysis of diastolic function be performed by the integrated approach of several techniques, the most important ones being: Pulsatile Doppler of the mitral flow, tissue Doppler of the mitral valve annulus, left atrial volume (LA) indexed by body surface and tricuspid regurgitation velocity.^20^ Pulmonary venous flow and Valsalva maneuver can be used as additional parameters in specific cases, which are useful in differentiating the degrees of diastolic dysfunction.^20^ While pulsatile and tissue Doppler velocities reflect the instantaneous filling pressures of the LV, the measurement of the LA volume reflects the cumulative effect of filling pressures over time and, therefore, this index is the chronic expression of diastolic dysfunction.^22^ However, it is important that other causes of LA enlargement are discarded and that this data is taken into consideration along with the patient’s clinical condition, chamber size and Doppler indices for the evaluation of diastolic function.

In individuals with preserved systolic function and without structural heart disease, diastolic dysfunction is considered if there is a change of more than 50% of the following 4 parameters: relationship between the early diastolic velocity of mitral inflow (E) and the early diastolic velocity of the mitral annulus (e’) E/e’ mean > 14; septal e´ velocity’ < 7 cm/s or lateral < 10 cm/s; tricuspid regurgitation velocity > 2.8 cm/s and LA indexed volume > 34 mL/m.^20,21^ For the group of patients with systolic dysfunction and those with preserved systolic function concomitant with the presence of cardiac disease (clinical or echocardiographic manifestation), the integrated use of the information allows us, in most cases, to estimate the ventricular filling pressures and the graduation of diastolic dysfunction.^20^ Three patterns of diastolic dysfunction are defined, in ascending order of severity: grade I (abnormal ventricular relaxation without increase of filling pressures); grade II (elevation of filling pressures coexisting with altered relaxation, usually presenting “pseudonormal pattern” of the mitral flow); and grade III (very high filling pressures, accompanied by a restrictive pattern of the mitral flow). To define the presence of increased filling pressures in this group with heart disease, we must first analyze the mitral flow, before other parameters. The relationship between E and the atrial diastolic velocity of the mitral inflow (A) E/A ≤ 0.8 (with E-wave ≤ 50 cm/s) is compatible with normal filling pressures and isolated abnormal relaxation, while the relation E/A ≥ 2 is consistent with elevated filling pressures. However, for cases with an E/A > 0.8 and < 2, an abnormalityof at least 2 of the following 3 parameters is required: E/e’; tricuspid refurgitation velocity; and LA indexed volume. In some cases, the definition criteria for diastolic dysfunction are not completely fulfilled, and thus the degree of diastolic dysfunction can be reported as indeterminate.^20^ This algorithm for the evaluation of diastolic dysfunction from the echocardiography has recently been validated in a multicenter study which assessed patients with and without left ventricular systolic dysfunction.^23^ Non-invasive evaluation of filling pressures by echocardiography correlated with the diastolic pressures measured by catheterization, showing greater accuracy than isolated clinical parameters.^23^

It should be noted that the parameters for evaluation of diastolic function may present important limitations in specific situations, such as hypertrophic cardiomyopathy, mitral annular calcification, severe mitral regurgitation, cardiac transplantation and cardiac arrhythmias.^20^ Some patients, even with grade I diastolic dysfunction defined at rest, become symptomatic only during exercise and therefore it may be useful to analyze filling pressures during physical stress (diastolic stress echocardiography).^20,24^ Patients with diastolic dysfunction are unable to increase ventricular relaxation with exercise, when compared to normal subjects, with increased filling pressures, which can be identified by increased E/e’ ratio and tricuspid regurgitation velocity.^24^ In normal patients, velocities of E and e’ increase proportionally and the index remains constant. Finally, the evaluation of diastolic function using techniques derived from strain and strain rate is promising, but requires further studies to establish its additional clinical value.^20^

### 2.3. Cardiomyopathies

Cardiomyopathies are a heterogeneous group of myocardial diseases associated with mechanical and/or electrical dysfunction, which usually exhibit inappropriate ventricular hypertrophy or dilatation, due to a variety of causes, often genetic.^25^ Cardiomyopathies are confined to the heart or are part of generalized systemic disorders. The classification is based on functional or structural changes in the following subtypes: dilated, hypertrophic, restrictive, and arrhythmogenic cardiomyopathy (or dysplasia) of the right ventricle (RV), more recently referred to as arrhythmogenic cardiomyopathy.^26^ Subsequently, as the knowledge on the genetics foundations of cardiomyopathies developed, other classifications have been proposed, subdivided into genetic, acquired and mixed.^26^ More recently, channelopathies and related disorders, such as long and short QT syndrome, Brugada syndrome and catecholaminergic polymorphic ventricular tachycardia, have been included in the group of cardiomyopathies, since they are cardiomyocyte diseases characterized by arrhythmogenic electrophysiological dysfunction.^25,26^

#### 2.3.1. Dilated Cardiomyopathy

It is characterized by LV dilation associated with global systolic dysfunction, in the absence of volume or pressure overload. The prevalence of dilated cardiomyopathy (DCM) is variable, reflecting the geographical and ethnic differences, as well as the methodologies used. A prevalence of 1:250 is estimated, based on the frequency of the left ventricular dysfunction as an expression of DCM.^27^ The criterion to define LV dilation is the final diastolic diameter > 2.7 cm/m^2^. With increased gradual dilatation on the short axis, the LV cavity becomes spherical, with sphericity index (long/short axis dimension) close to 1 (normal value > 1.5).^28^ Wall thickness is usually normal, but the myocardial mass is increased. The degree of impairment of systolic function is variable, and systolic dysfunction is often progressive. LV volumes are calculated in a more reproducible and accurate way by using the 3D echocardiography. Abnormalities associated with diastolic function may be present, contributing to the variation in the clinical and hemodynamic presentation of DCM. The involvement of the RV can be evidenced, but it is not a criterion for the diagnosis of DCM.^29^ Notably, DCM is associated with an increased risk of severe arrhythmia, indicating the pathological involvement of the cardiac conduction system. Complex remodeling of one or both ventricles contributes to the secondary features of DCM, which include functional mitral and tricuspid regurgitation, enlarged atria, intracavitary thrombi, and evidence of low cardiac output.^28^ In the context of DCM, the analysis of diastolic function aims to estimate the filling pressures; and the mitral flow pattern is usually enough to identify patients with increased LA pressure. E-wave deceleration time is an important predictor of outcomes in these patients.^30^ Other diastolic dysfunction parameters, including the E/e’ ratio, have good correlation with pulmonary capillary pressure and have an additional prognostic value for LVEF.^30^

Echocardiography is the imaging method of choice for the evaluation of DCM patients, providing key data not only for diagnosis, risk stratification and treatment definition, but also plays a key role in the evaluation of family members.^28^ Key echocardiography indications in the evaluation of DCM are displayed in table 1. Transthoracic echocardiography (TTE) is indicated in the initial evaluation of patients with heart failure and suspected DCM. TTE is recommended in first-degree relatives of DCM patients due to the high incidence (20 to 50%) of familial DCM.^28^ Several echocardiographic parameters were used to assess mechanical desynchrony in patients with DCM. However, the broader role of echocardiography in the selection of patients for cardiac resynchronization therapy remains undefined. Currently, echocardiographies are limited to patients with borderline QRS duration (120 to 149 ms), whose presence of intra- or interventricular desynchrony may provide additional information.^28^ The echocardiography guiding the placement of electrodes at the site of greater mechanical activation delay (evaluation by speckle tracking) showed benefit in heart failure free survival, with a more favorable impact on ischemic heart disease compared to DCM.^31^

**Table 1:** Recommendation of echocardiographies in dilated cardiomyopathies

Recommendation	Class of recommendation	Level of evidence
Assessment of patients with suspected dilated cardiomyopathy or heart failure	I	C
Assessment of signs and symptoms suggestive of myocardial dysfunction	I	C
Reassessment of patients with cardiomyopathy known to present worsening of symptoms or to require changes in therapy	I	C
First-degree relatives of patients with dilated cardiomyopathy	I	B
Assessment of candidate patients for cardiac resynchronization therapy with LBBB and QRS duration between 120 and 149 ms	IIa	C
Reassessment of routine in patients with stable dilated cardiomyopathy, without clinical or therapeutic changes	III	C
**Chagasic cardiomyopathy**		
Initial evaluation of patients with positive serology for Chagas disease for diagnosis and risk stratification of cardiomyopathy	I	C
Patients with the indeterminate form of Chagas disease who present new electrocardiographic alterations compatible with the development of cardiomyopathy	I	C
Patients who present worsening symptoms of heart failure, syncope, arrhythmic or thromboembolic events	I	C
Routine reassessment of clinically stable patients with no changes in therapy	III	C

LBBB: left bundle branch block

#### 2.3.2. Chagasic Dilated Cardiomyopathy

Chagasic dilated cardiomyopathy (CCM) presents similar characteristics to idiopathic DCM, but with predominance of segmental changes in contractility, especially in the basal segments of the inferior and inferolateral walls.^32^ Apical aneurysm is a typical CCM finding and is useful in the differential diagnosis of dilated cardiomyopathies.^33^ The morphology of aneurysms is variable and non-standard sections are often required for the identification of apical contractile changes. The presence of thrombi within the aneurysms is frequent and associated with cerebral thromboembolic events.^34^ Diastolic dysfunction is universally present in patients with CCM and heart failure.^35^ The main echocardiographic parameters previously studied with a prognostic value in CCM are LVEF, right ventricular function, LA volume and E/e’ ratio.^33,36^ The contractile function of the LA evaluated by the negative peak of the global atrial strain was an independent predictor of clinical events in the CCM.^35^ The heterogeneity of systolic contraction, quantified by mechanical dispersion to speckle tracking, was associated with ventricular arrhythmias in patients with CCM, regardless of LVEF.^37^ Recommendations for performing TTE in CCM are set out in table 1.^38^

#### 2.3.3. Cardiac Resynchronization Therapy and Pacemaker Optimization

Cardiac resynchronization therapy is an established treatment option for patients with heart failure with marked reduction of LVEF. The echocardiography is fundamental in the indication, estimation of success and evaluation of the results of this procedure and may also contribute to the recovery of unfavorable results. This treatment is indicated as class I in patients with heart failure (New York Heart Association (NYHA) functional class II, III or IV), with LVEF lower than 35%, optimized medication and left bundle branch block with QRS duration above 150 ms.^28^ Also in class IIa and IIb indications, it is necessary to recognize the reduction of the LVEF below 35%, being contraindicated when this value is not present. Therefore, in the possibility of indication for cardiac resynchronization therapy, transthoracic echocardiography is a class I indication, level of evidence C. In this examination, it is mandatory that LVEF be obtained by the Simpson 2D method, with a description, in the report, of their volumes. It is also possible to use the 3D methodology, of less variability, although still unproven in this clinical scenario. Approximately 30% of patients do not present clinical improvement or significant reduction in LV final systolic volume.^39^ TTE can provide information that helps identify a greater probability of successful treatment response, such as the presence of mechanical, inter- and intraventricular desynchrony, the presence of myocardial reserve and determination of the last site of activation, which may be associated with higher degree of fibrosis. To this end, the use of a variety of methods is encouraged, from the visual evaluation of 2D echocardiography, M-mode,^40^ tissue Doppler and, especially, the use of a technique that evaluates longitudinal^41^ or radial myocardial deformation.^42,43^ In the evaluation of successful response to treatment, it is expected, in regards to imaging, mainly negative remodeling to be observed, usually characterized by reduction of 15% of the initial systolic volume, analyzed between 3 and 6 months of the implant.^39,44^ In case the negative remodeling and/or the clinical improvement of the patient is not met, one possibility is to adjust the pacemaker, guided by TTE, to optimize the atrial and ventricular stimulus intervals. The main correction in this case seems to be adjustment of the atrial and ventricular stimulus intervals, guided by the echocardiography, which allows for retrieval of the results.^45,46^

#### 2.3.4. Assessment after Heart Transplantation

**Figure 1:**
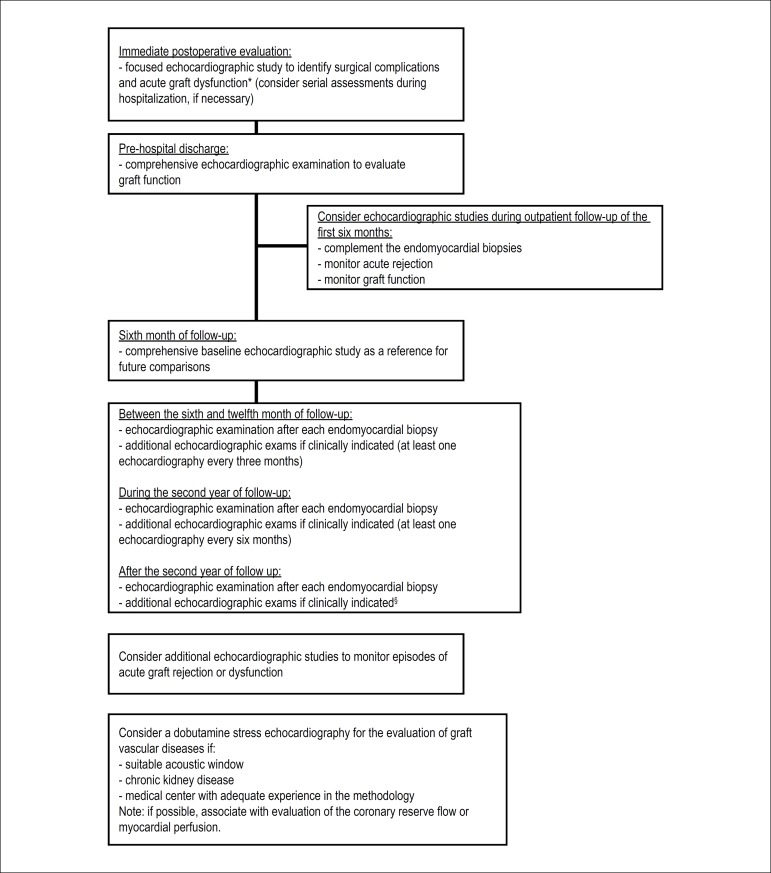
Echocardiographic evaluation after cardiac transplantation. ^*^Graft dysfunction: confirmed on echocardiographic examination by dropping the ejection fraction by more than 10% to a value lower than 50%, compared to the baseline examination of the sixth month; ^§^patients with graft dysfunction suspected or confirmed; clinical symptoms of a possible new cardiac abnormality; alterations in the resting electrocardiogram.

The echocardiography is the main noninvasive imaging modality as well as the most versatile one in the evaluation and monitoring of patients after cardiac transplantation, providing accurate information on morphology and graft function. From the immediate postoperative period up to the moment of hospital discharge, serial echocardiographic exams are recommended both to identify and monitor surgical complications and early graft dysfunction, whether due to primary or secondary causes (e.g., reperfusion injury, non-responsive pulmonary hypertension (class I, level B).^47,48^ In the presence of early graft dysfunction, the echocardiography usually shows an overall reduction in myocardial function (LVEF < 45%), loss of the contractile reserve, increase in RV volume with systolic dysfunction (tricuspid annular plane systolic excursion - TAPSE < 15 mm or RV ejection fraction < 45%).^47^ A comprehensive echocardiographic examination (class I, level B) is recommended in the sixth month after cardiac transplantation), which will serve as the baseline for assessing graft morphology and function during sequential and regular follow-up examinations (interval and frequency of exams in figure 1).^47^ Quantifications of cardiac chambers size and volumes, RV systolic function, LV diastolic and systolic parameters, and pulmonary arterial pressure should be performed on the sixth month and subsequent echocardiographies.^47^ It is recommended that such echocardiographic studies also include advanced methodologies, such as the study of myocardial deformation (strain) and 3D evaluation of the volumes and function of cardiac chambers and tricuspid valve (frequently injured during the endomyocardial biopsy procedure), for providing a more accurate and comprehensive analysis (class I, level B).^47^

It should be noted that there is no single isolated echocardiographic parameter that can be reliably used to diagnose acute rejection.^47^ However, an echocardiographic study with no change from the baseline study has a high negative predictive value for acute rejection of the graft. On the other hand, if several echocardiographic parameters are abnormal, the probability of acute rejection of the graft increases considerably.^47^ When an abnormality is detected, a careful review of the images of the present study and the baseline study (side-by-side) is highly recommended (class I, level B).^47^ GLS is an adequate parameter to assist in the subclinical diagnosis of graft dysfunction, regardless of etiology, in addition to an adverse event predictor, when comparing the variations of values occurred during serial evaluations (class IIb, level B).^47,49,50^ The association of GLS with endomyocardial biopsy helps to characterize and monitor an episode of acute rejection or global dysfunction.^47^ Pericardial effusion should be serially assessed for extent, location and hemodynamic impact (class IIb, level B). In the case of a recently detected pericardial effusion, the hypothesis of acute rejection should be considered, taking into account the patient’s global and clinical echocardiographic evaluation.^47,51^ Cardiac graft vascular disease is the main cause of late complication; and the dobutamine stress echocardiography has proven to be a safe and accurate method to identify the affected patients.^47,52-54^ The evaluation of the coronary reserve flow, as well as the sonographic contrast infusion to highlight the borders and to evaluate the myocardial perfusion , when combined with stress echocardiography, have been shown to increase the accuracy of the diagnosis of graft vascular disease.^55-59^ Thus, dobutamine stress echocardiography alone (class IIA, level B) or in association with the evaluation of the flow of coronary reserve and/or with the use of sonographic contrast (class I, level B) may be an adequate noninvasive alternative to routine coronary angiography to assess the presence of cardiac graft vasculopathy, provided that the medical center has good experience with methodologies.

In addition to the role of cardiac graft monitoring, intraoperative echocardiography can be used as an alternative to fluoroscopy to guide endomyocardial biopsies, avoiding repeated exposure to X-rays, particularly in children and young women (class I, level B). Whether in transthoracic or transesophageal mode, the echocardiography allows a simultaneous visualization of the soft tissues and the biotope, guaranteeing greater biopsy safety in different regions of the RV with a reduction in the complication rate.^47,60^ Furthermore, the use of echocardiography during the procedure allows immediate recognition and management of a possible complication.

#### 2.3.5. Monitoring of Cardiac Function During Chemotherapy with Cardiotoxic Drugs

Current cancer therapy is quite effective in some types of tumors, though it can induce cardiovascular complications. Cardiotoxicity (CT) induced by cancer treatment is recognized as the major cause of morbidity and mortality in cancer survivors.^61^ Before starting anti-neoplastic treatment, it is essential to access the risk of CT,^62^ taking into consideration: (a) the specific risk of the drug used in chemotherapy, as some of them may affect the cardiac function (anthracyclines, trastuzumab), while other ones, the vascular function (5-fluoracil, capecitabine), or both (bevacizumab); (b) the use of radiotherapy, as it increases the risk of heart failure when concomitant with anthracyclines, pericardial lesion (constrictive pericarditis) and coronary artery disease; (c) the presence of previous risk factors, such as age > 65 years, female gender, hypertension, diabetes mellitus, coronary artery disease and history of heart failure. All patients receiving potentially cardiotoxic drugs should be periodically monitored for CT signs, which can be classified according to the injury the drug used produces.^63^ CT Type I, potentially irreversible, anthracycline-related dose, is dose-dependent, mainly at > 250 to 300 mg/m^2^ (often used in the treatment of breast cancer, lymphoma, leukemia, and sarcoma). It most commonly occurs in the first year of chemotherapy, or even two to three decades after completion of treatment, as progressive systolic dysfunction. It may rarely present as an acute systolic dysfunction, immediately after dosing. Type II CT, which is potentially reversible, mainly related to trastuzumab (used in the treatment of breast cancer in patients with increased HER2 receptor expression), has no relation to the cumulative dose.^63^ Such information is the basis for the algorithms of serial left ventricular function monitoring during and after treatment of cancer patients, published by the European Association of Cardiovascular Imaging (EACVI) and the American Society of Echocardiography (ASE).^3^ The most historically used parameter is LVEF, calculated by TTE using the Simpson biplanar 2D method.^4^ LVEF values ​​between 53 and 73% should be considered normal in the evaluation. The main advantages of 2D TTE in relation to other imaging modalities, such as radioisotope ventriculography and magnetic resonance imaging (MRI), are: greater availability, lower cost, possibility of serial re-evaluations and greater safety (absence of radiation and limitation in patients with renal insufficiency). 3D TTE, used in sequential and comparative MRI evaluations for LVEF assessment, showed reproducibility comparable to MRI and better accuracy than 2D TTE,^64^ being more indicated, when available, in the serial evaluation of these patients.^65^

The definition of CT due to chemotherapy was defined by the consensus of these two societies^3^ as the decrease of LVEF > 10 percentage points to values < 53% and should be confirmed after 2 to 3 weeks of diagnosis by new imaging. This decrease may or may not be accompanied by symptoms of heart failure and may or may not be reversible. One of the major limitations of the use of LVEF for CT diagnosis in the follow-up of these patients is that changes in LVEF occur later. In order to minimize the risk of developing irreversible cardiomyopathy, it is essential to identify early signs of CT, since the administration of cardioprotective medication in this phase may result in an improvement in cardiac function.^66^ Thus, the search for a technique that allows subclinical and early detection of CT before LVEF decrease or the onset of clinical symptoms has been an area of intense investigation. In this scenario, the use of GLS gained importance, evaluating myocardial deformation. Such technique has inter- and intraobserver reproducibilities smaller than the LVEF obtained by the 2D TTE, but is limited by the variability of normal values ​​according to the brand of the equipment used, age and gender of the patients.^67^ Systematic review confirmed the prognostic value of the alterations in GLS for CT, preceding the LVEF decrease obtained by 2D or 3D TTE.^15^ The consensus recommends serial GLS evaluation in patients at risk of CT, with subclinical left ventricular dysfunction suggestive of a fall of > 15% of the baseline value, even without LVEF change.^63^ The relative decrease between 8 and 15% suggests a more rigorous follow-up. GLS Variation of < 8% is consistent with absence of subclinical dysfunction.^63^ Although some studies have drawn attention to changes in diastolic function following chemotherapy,68 there is no current evidence to support such parameters as indicative of CT.^63^ A use of biomarkers in the assessment integrated with imaging methods in chemotherapy patients evidenced the importance of troponin I (TnI) with a high negative predictive value in the detection of CT.^69^ It is probable that patients who do not evolve with TnI elevation have lower probability of events and perhaps less need for imaging tests in subsequent evaluations.^69,70^ There is still no robust scientific evidence based on randomized clinical trials to support the algorithms proposed by the European Society of Oncology^71^ and the consensus of EACVI and ASE,^63^ in the follow-up of these patients; however, these documents represent current knowledge in the area. The orientation of the EACVI - ASE consensus^63^ to the present moment is:

- Initial evaluation of left ventricular function before the start of chemotherapy in patients who will use potentially cardiotoxic chemotherapeutics. If it is not possible in all patients, it is recommended in those at high risk for the development of CT: age > 65 years, previous left ventricular dysfunction, predicted use of high doses of anthracyclines (type I) or combination of type I and II drugs. To perform the LVEF assessment by the 3D TTE if available or, alternatively, by the 2D TTE (Simpson method). It is desirable that the evaluation by GLS and TnI is carried out. If not possible to perform GLS, report the S-wave of the medial and lateral tissue Doppler of the mitral annulus. Further monitoring of left ventricular function is recommended after this initial evaluation, depending on the chemotherapy to be initiated.
- Type I drugs (anthracyclines): evaluate left ventricular function (2D/3D LVEF and GLS) at the end of chemotherapy and after 6 months at dose < 240 mg/m^2^. For doses > 240 mg/m^2^, evaluate left ventricular function before each additional 50 mg/m^2^ cycle at the end of chemotherapy and after 6 months.
- Type II drugs (trastuzumab): evaluate left ventricular function every three months during chemotherapy.
- Patients receiving trastuzumab following anthracycline treatment: assess left ventricular function every three months during chemotherapy and six months after its completion.

#### 2.3.6. Hypertrophic Cardiomyopathy

Hypertrophic cardiomyopathy (HCM) is a genetic cardiovascular disease characterized by increased left ventricular wall thickness ≥ 15 mm in adults, with non-dilated ventricular cavity not explained by abnormal loading conditions, such as arterial hypertension or valve aortic stenosis.^72^ Minor hypertrophy degrees (13 to 14 mm) may also diagnose HCM, particularly among relatives of these patients. TTE is considered the initial imaging for diagnosis, stratification of the risk of cardiac events and management of patients with HCM. Among the parameters to be evaluated in HCM are: location and hypertrophy degree; identification of obstruction and intraventricular gradient at rest or intentionally caused; presence of magnitude of mitral reflux; systolic and diastolic function; and LA size. Any hypertrophy pattern may be found, though the asymmetric is the most frequent one (75% of cases), and it is more common at the confluence of the anterior interventricular septum with the LV free wall.^73^ Other forms of hypertrophy are: basal, concentric, apical and lateral wall. There is a linear association between maximum myocardial thickness and sudden death, with greater risk in patients with thickness ≥ 30 mm.^72,74^ Gradual identification of the LV outflow tract is important in the management o symptoms and in the stratification of the risk of sudden death.^72^ TTE evaluation generally characterizes the presence of LV outflow tract obstruction (instantaneous gradient ≥ 30 mmHg) at rest (one third of patients) of after provocative maneuvers (one third), such as exercises (echocardiography under physical stress) or Valsalva maneuver. Echocardiography under physical stress can be very useful in patients with HCM, since in addition to detecting the presence and degree of obstruction during effort, it allows the objective evaluation of symptoms, functional capacity, systolic blood pressure response and the presence of secondary mitral regurgitation. Approximately 25% of HCM patients have abnormal blood pressure response during exercise, characterized by a drop in systolic pressure or by failure to increase in > 20 mmHg. This finding has been interpreted as a risk factor for unfavorable prognosis and sudden death.^75^ Stress echocardiography with dobutamine is not recommended. The cut-off value of the intraventricular gradient ≥ 50 mmHg at rest or after provocative maneuvers is considered when indicating surgical treatment or percutaneous intervention in symptomatic patients, despite therapy with optimized medication.^72^ Patients with HCM in general have diastolic dysfunction, commonly with altered relaxation (grade I), though without significant correlation between mitral flow data and LV filling pressures. Thus, the integrated approach of mitral Doppler data, tissue Doppler, pulmonary vein flow and LA volume is recommended in these patients.^76^ LVEF is normal or increased in most patients, giving the false impression of preserved systolic function. However, longitudinal strain assessment invariably shows a global and regional decrease (coincident with sites of greater hypertrophy) of contractility.^77^ Estimating the size of the LA is fundamental, as there is a significant correlation between the dilation of the chamber and an increased risk of cardiovascular events, such as atrial fibrillation and sudden death. The main complication of HCM is sudden cardiac death (SCD), especially in young and apparently healthy individuals.^78,79^ Cardiac defibrillator implantation for primary or secondary prophylaxis may reduce mortality from this complication and is the only therapy with evidence of life-saving potential.^80^ TTE has a relevant role in the two most commonly used risk stratification scores form SCD in HCM, which determined the relationship between come clinical risk factors and prognosis. In the American model of primary prevention, one of the risk factors among 5 variables is the presence of interventricular septum thickness ≥ 30 mm.^75^ In the European model, of the seven variables analyzed, three of them are provided by TTE: septum thickness, LA and left ventricular outflow tract (LVOT) gradient at rest or after Valsalva maneuver.^72^ Family screening of first-degree relatives of HCM subjects should be performed periodically due to their risk of developing the disease. Recommendations for the use of echocardiography in HCM are summarized in table 2.

**Table 2:** Recommendations of transthoracic echocardiography, echocardiography under physical stress and transesophageal echocardiography in hypertrophic cardiomyopathy^72,75^

Recommendation	Class of recommendation	Level of evidence
TTE in the initial assessment of all patients with suspected HCM, at rest and during Valsalva maneuver	I	B
EPS in symptomatic patients with resting or Valsalva intraventricular gradient < 50 mmHg to assess the degree of dynamic obstruction and mitral regurgitation during exercise	I	B
Reassessment by TTE when there are changes in symptoms or a new cardiovascular event	I	B
TTE in the assessment of therapeutic results of pharmacological, surgical (myomectomy), interventional (alcoholic septal artery occlusion) and pacemaker treatments	I	C
TTE in the screening of first-degree relatives with HCM diagnosis	I	B
Serial TTE (every 12 to 18 months) in children of HCM patients, starting at age 12 (or earlier, if there is intention to take on competitive sports or sudden death among relatives)	I	C
TTE during alcohol septal artery ablation	I	B
TEE in intraoperative myectomy monitoring and during alcoholic occlusion of the septal artery with inadequate TTE	I	B
Serial TTE every one to two years may be useful in stable symptomatic patients to reassess myocardial hypertrophy, dynamic obstruction, and ventricular function	IIa	C
TEE may be useful when TTE is inconclusive, in the planning of myomectomy or in the evaluation of mitral regurgitation secondary to mitral valve abnormalities	IIa	C
Serial TTE (every five years) is reasonable in periodic reassessment in first-degree relatives of adult HCM patients	IIa	C
TTE combined with intravenous contrast injection is reasonable if the diagnosis of apical HCM and/or apical infarction are doubtful, or the quantification of hypertrophy is inadequate, especially if MRI is unavailable, non-diagnostic or contraindicated	IIa	C
EPS may be useful in asymptomatic HC patients, with no dynamic obstruction at rest when gradient detection in LVOT is relevant for lifestyle or career change orientation, or decision making on medical treatment	IIb	C
TTE should not be performed in less than 12 months in HCM patients when there is no change in symptoms or predicted conduct change	III	C

TTE: transthoracic echocardiography; HCM: hypertrophic cardiomyopathy; EPS: echocardiography under physical stress; TEE: transesophageal echocardiography; LVOT: left ventricular outflow tract

#### 2.3.7. Restrictive Cardiomyopathies

Restrictive cardiomyopathies (RCM) are a group of entities characterized by abnormalities in the ventricular filling pattern, which may be associated with thickened and rigid walls and generally preserved systolic function. RCMs comprise various entities, including, endomyocardial fibrosis (EMF), endomyocardial fibroelastosis, Löefler parietal endocarditis, infiltrative (such as amyloidosis and sarcoidosis), storage (such as hemochromatosis and Fabry disease), idiopathic and other forms secondary to different processes (scleroderma, carcinoid syndrome, metastases of systemic neoplasms, anthracycline toxicity and irradiation heart disease).^81^ Diagnosis by echocardiography is based on common anatomical and functional changes: ventricular cavities of normal or reduced size, usually with Doppler degree III diastolic function (restrictive type), generally preserved overall systolic function, and dilated atria. Tissue Doppler analysis shows velocity e’ obtained in the septal mitral annulus usually below 7.0 cm/s, a useful measure in constrictive pericarditis differentiation.^82^ In amyloidosis, there is thickening of the atrioventricular valves, myocardial walls, and eventually the atrial septum, with a more intense (ecorrefringence) reflection and “granular and sparkling” myocardial aspect.^83^ The GLS analysis of the LV with 2D echocardiography in amyloidosis observes very low values, especially in the mid and basal segments with relative “apical sparing” (it aids in the differential diagnosis with other diseases).^84^ In EMF, one may observe: obliteration of the apex by fibrosis, signs of ventricular restriction, and involvement of the atrioventricular valves. The fibrosis of the apical thrombi is differentiated due to absence of akinesia or dyskinesia in the left EMF. Another differential diagnosis is apical HCM, which presents no endocardial thickening or restrictive pattern and displays specific electrocardiographic changes. Cardiac sarcoidosis may present with regional contractile abnormalities and non-ischemic distribution aneurysms. GLS measure represents an early marker of myocardial involvement in sarcoidosis and the magnitude of the reduction is associated with poor prognosis.^85^ The recommendations for TTE in RCM are set out in table 3. Transesophageal echocardiography (TEE) is indicated when there are technical difficulties to TTE and in the transoperative monitoring of fibrosis and apical correction of valve defects.

**Table 3:** Recommendations of echocardiography in restrictive cardiomyopathies

Recommendation	Class of recommendation	Level of evidence
Diagnostic investigation of patients with heart failure without clear etiology	I	C
Differential diagnosis of patients with restrictive syndrome	I	C
Symptomatic patients with systemic diseases potentially causing RCM	I	C
Patients with hypereosinophilic syndrome, ascites and distended jugular veins	I	C
Patients with ascites and lower limb edema, without established diagnosis	I	C
Patients submitted to radiotherapy with signs of systemic venous hypertension	I	C
Reassessment of patients with previous RCM diagnosis when there is a change in the clinical course of the disease	I	C
Patients with EMF for therapeutic planning and prognostic evaluation	IIa	C
Patients with edema and ascites, with evidence of normal systemic venous pressure and no evidence of cardiopathy	III	C

RCM: restrictive cardiomyopathies; EMF: endomyocardial fibrosis.

#### 2.3.8. Arrhythmogenic Cardiomyopathy (Right Ventricle Arrhythmogenic Dysplasia)

Arrhythmogenic cardiomyopathy (AC) is considered an inherited cardiomyopathy with autosomal dominant transmission, predisposing to the emergence of ventricular arrythmias, sudden death in young people, ventricular dysfunction and heart failure. Due to the frequent involvement of the LV, the use of the term CA is currently recommended, which comprises both ventricles, replacing the term “arrhythmogenic dysplasia of the RV”.^86^ The disease is characterized by a progressive replacement of the ventricular myocardium by fibrous and adipose tissue, which can lead to thinning of the wall and aneurysm formation. In the RV, the process is typically located in the inferior, apical and infundibular walls (dysplasia triangle), and may be diffuse or segmental. LV involvement occurs in more than half of the cases, typically located in the subepicardium or mesocardium, and often confined to the inferolateral segment. Echocardiography is the imaging modality of choice in the initial assessment of AC (Table 4) and the most commonly used propaedeutic method for patient follow-up.^87^ Typical morphologic features in patients with CA include regional contractile abnormalities and/or dilation and right ventricular dysfunction. Among the traditional echocardiographic criteria, derived from 2D echocardiography, proposed for the diagnosis of AC,^88^ are: presence of akinesia, dyskinesia or right ventricular aneurysm; increased ventricular outflow tract diameter (measured on long and short parasternal axis); and reduction of the fractional variation of the RV area. Recently, the routine and systematic addition of other echocardiographic measurements and techniques was recommended in order to improve evaluation:^89^

**Table 4:** Recommendations of the echocardiography in arrhythmogenic cardiomyopathy

Recommendation	Class of recommendation	Level of evidence
Assessment of patients with suspected AC	I	B
Reassessment of patients with known AC when there is change of symptoms or new cardiovascular event	I	C
Family screening in first-degree relatives of AC patients	I	C
Routine re-evaluation of clinically stable patients with no changes in therapy	III	C

AC: arrhythmogenic cardiomyopathy

- Conventional parameters: basal RV diameter (normal ≤ 41 mm); systolic excursion of the annular tricuspid plane (TAPSE - normal ≥ 17 mm).
- Advanced parameters: wave s’ to the tissue Doppler of the RV’s free wall (normal ≥ 9.5 cm/s); longitudinal strain of the RV’s free wall (normal ≥ -20%); LV’s GLS (normal ≥ -18%); RV ejection fraction to 3D echocardiography (normal ≥ 45%).

In short, TTE, preferably with analysis of conventional and advanced parameters, is indicated in patients with suspected or established AC (evaluation of disease progression), as well as in family screening of first-degree relatives.

#### 2.3.9. Non-compaction Cardiomyopathy

Non-compaction cardiomyopathy (NCC) is considered a distinct cardiomyopathy, marked by genetic heterogeneity, with an overlapping of different phenotypes and great variability of clinical presentation. As a consequence, there is still controversy in the literature regarding its nomenclature: whereas for the American Heart Association (AHA)^90^ it is considered a primary cardiomyopathy, the European Society of Cardiology (ESC)^81^ considers it an unclassified disorder. Its pathogenesis implies early interruption of compaction of the trabecular meshwork of the LV during embryogenesis, resulting in the formation of two layers: a thin compacted epicardial layer and a thick endocardial one (similar to a “spongy” mesh) with marked trabeculations and deep intertrabecular recesses. The 2D echocardiography is the basis for diagnosis, follow-up, and better delineation of NCC phenotypic expressions.^91^ Several criteria have been employed in diagnosis, taking into account the increase in the proportion of the non compacted layer (for example, the non compacted/compacted ratio at the end of systole > 2), presence of excessive trabeculation, hypokinesia of non compacted areas (commonly located at the apex and lateral wall) and visualization of flow in the recess (via color Doppler). New techniques have recently been incorporated to aid in diagnosis, such as the use of echocardiographic contrast, 3D echocardiography and myocardial strain for the analysis of regional deformation and rotation (which assumes a characteristic pattern in this nosological entity).^92^ Therefore, it should be noted that the diagnosis of suspected cases has increased in recent years, due to advances and improvements in imaging methods, as well as the perception of the need to actively investigate first-degree relatives affected by the disease (described in 13 to 50% of this specific group).^93^ On the other hand, increasingly frequent “exam findings” (physiological versus pathological variants) have been reported in clinical practice, leading to the worrisome excess of diagnoses.^7^ Therefore, it is recommended to carry out a comprehensive evaluation, including clinical, electrocardiographic data and careful analysis of the findings in complementary imaging studies.^94^ The recommendations for performing the echocardiography in NCC are set out in table 5.

**Table 5:** Recommendations of echocardiography in non-compaction cardiomyopathy

Recommendation	Class of recommendation	Level of evidence
NCC clinical suspicion	I	C
Reassessment of patients with known NCC when there is change of symptoms or new cardiovascular event	I	C
Screening in first-degree relatives of NCC patients	I	C
Carriers of muscular diseases and/or other clinical syndromes that may be related	I	C
Use of new techniques such as strain, 3D echocardiography and echocardiographic contrast for complementary evaluation and aid in differential diagnosis	IIa	B
Routine reassessment of clinically stable patients with no change in therapy	III	C

NCC: non-compaction cardiomyopathy

### 2.4. Arterial Hypertension and Myocardial Hypertrophy

The elevation of systolic stress in the LV wall, secondary to systemic arterial hypertension (SAH), can produce myocardial hypertrophy by increasing ventricular mass.^95^ Unlike physiological hypertrophy (growth, pregnancy and physical activity), characterized by preserved cardiac structure and function, left ventricular hypertrophy (LVH), secondary to SAH, is commonly associated with fibrosis, myocardial dysfunction and increased mortality.^96^ The echocardiography is the clinical choice exam to detect LVH, due to its being more accurate than the electrocardiogram^97,98^ and allowing estimation of LV mass (LVM). The methodology to measure LVM and to define its cut-off points and index form (body surface, height, weight) varies between studies. Most echocardiography authors and laboratories follow the recommendations published by ASE and EACVI.^95,99^ LVM indexing to the body surface area in g/m^2^ is the most used one,^100^ and normality values are different for men and women (Table 6).^95,99^

**Table 6:** Degree of abnormalities of left ventricular mass^95,99^

Linear method	Female				Male			
	Normal	Slight increase	Moderate increase	Severe increase	Normal	Mild increase	Moderate increase	Severe increase
LV mass, g	67 to 162	163 to 186	187 to 210	≥ 211	88 to 224	225 to 258	259 to 292	≥ 293
Mass/BS, g/m^2^	43 to 95	96 to 108	109 to 121	≥ 122	49 to 115	116 to 131	132 to 148	≥ 149
Mass/height, g/m	41 to 99	100 to 115	116 to 128	≥ 129	52 to 126	127 to 144	145 to 162	≥ 163
Mass/height^2,7^, g/m^2,7^	18 to 44	45 to 51	52 to 58	≥ 59	20 to 48	49 to 55	56 to 63	≥ 64
RWT (2 x LVPW/LVDD)	0.22 to 0.42	0.43 to 0.47	0.48 to 0.52	≥ 0.53	0.24 to 0.42	0.43 to 0.46	0.47 to 0.51	≥ 0.52
Septum thickness, cm	0.6 to 0.9	1.0 to 1.2	1.3 to 1.5	≥ 1.6	0.6 to 1.0	1.1 to 1.3	1.4 to 1.6	≥ 1.7
LVPW thickness, cm	0.6 to 0.9	1.0 to 1.2	1.3 to 1.5	≥ 1.6	0.6 to 1.0	1.1 to 1.3	1.4 to 1.6	≥ 1.7
**2D Method**								
LV mass, g	66 to 150	151 to 171	172 to 182	≥ 193	96 to 200	201 to 227	228 to 254	≥ 255
Mass/BS, g/m^2^	44 to 88	89 to 100	101 to 112	≥ 113	50 to 102	103 to 116	117 to 130	≥ 131

LV: left ventricle; BS: body surface; RWT: relative wall thickness; LVPW: left ventricle posterior wall; LVDD: left ventricle diastolic diameter; 2D: two-dimensional

Cumulative exposure to elevated blood pressure levels among young adults is associated with LV systolic dysfunction in mid-life.^101^ The presence of LVH is considered as evidence of target organ damage in hypertensive patients, and its association with cardiovascular diseases and mortality is well documented.^102-104^ Such an increase in cardiovascular risk in hypertensive patients is directly related to LVM, regardless of blood pressure values.^103^ In addition to LVM, the geometric pattern of LVH is also seen as an important variable related to cardiovascular risk. Four patterns of LV geometry^99^ are described in (Table 7). The altered geometric patterns (concentric LVH, eccentric LVH and concentric remodeling) are predictors of cardiovascular complications in hypertensive patients, with concentric LVH being associated with higher risk of events.^103^

**Table 7:** Geometric patterns of the left ventricle^99^

Left ventricle geometry	Left ventricle mass /body surface (g/m^2^)	Left ventricle mass /body surface (g/m^2^)
Normal	≤ 115 (men) or ≤ 95 (women)	≤ 0.42
Concentric hypertrophy	> 115 (men) or > 95 (women)	> 0.42
Eccentric hypertrophy	> 115 (men) or > 95 (women)	≤ 0.42
Concentric remodeling	≤ 115 (men) or ≤ 95 (women)	> 0.42

*Measures taken by the linear method

Another frequent finding in SAH is the presence of LV diastolic dysfunction.^101^ Hypertensive individuals with heart failure commonly present with LVH, abnormalities in diastolic function and preserved ejection fraction. In these cases, diastolic dysfunction alone may be responsible for the signs and symptoms of heart failure.^105^ In addition, E/e’ ratio > 13 is associated with high cardiac risk in hypertensive patients, regardless of LVM.^106^ The use of GLS, obtained by 2D speckle tracking, allows for the early identification of subclinical systolic dysfunction in several scenarios, including hypertensive patients without LVH.^107^ GLS decline was related to hospitalization by heart failure, infarction, stroke, and death in patients with asymptomatic hypertensive heart disease.^108^ The regression of LVH in hypertensive patients, evaluated by serial echocardiographies after therapeutic interventions, is associated with decreased risk of fatal and non-fatal cardiovascular events, even in those cases where LVH has not been detected by the electrocardiogram.^109^ This benefit is directly related to the degree of reduction of LVM indexed to body surface, regardless of ambulatory blood pressure. LVH regression is also associated with an improvement in LV systolic^110^ and diastolic function^111^ in hypertensive patients. The thoracic aorta is more frequently affected by dilatation in hypertensive patients without adequate blood pressure control than in normotensive and controlled hypertensive ones.^112^ Long-term follow-up has shown that blood pressure levels are one of the main modifiable factors of adult aortic root dilatation.^113^ The recommendations for performing the echocardiography in SAH are listed in table 8.

**Table 8:** Recommendations of the echocardiography in the evaluation of hypertensive patients

Recommendation	Class of recommendation	Level of evidence
LVH detection	I	A
Assessment of systolic and diastolic function in hypertensive patients with clinical suspicion of heart failure	I	A
Hypertensive patients with left bundle branch block	I	C
Assessment of the aortic diameter in hypertension without adequate blood pressure control	I	B
Hypertensive patients with LVH on ECG for quantification of LVH and definition of LV geometric pattern	IIa	B
Global longitudinal strain evaluation in patients with hypertensive cardiopathy	IIa	C
Reassessment of patients with hypertensive heart disease without alteration of their clinical status	IIb	B
Assessment of first-degree relatives of hypertensive patients	III	C
Selection of antihypertensive therapy	III	C
Monitoring of antihypertensive therapy in controlled and asymptomatic individuals	III	C

LVH: left ventricular hypertrophy; ECG: electrocardiogram; LV: left ventricle

### 2.5. Athletes

The clinical entity called “athlete’s heart” has been recognized for more than two decades^114^ and is characterized by cardiac morphological alterations, mainly of increased ventricular mass, secondary to physical training stimulus. These alterations are not accompanied by changes in myocardial function, not only by conventional echocardiographic methods but also by techniques such as tissue Doppler and strain.^115,116^ Still, as a result of intact ventricular function, there is no significant increase in atrial cavities^117^ and reversibility of morphological alterations after discontinuation of training may be a decisive diagnostic factor in doubtful cases. The use of TTE, therefore, can elucidate cases of doubtful diagnosis between this situation and hypertrophies or pathological ventricular remodeling, such as HCM or even hypertrophy secondary to SAH.^115^ However, the use of echocardiography as a routine method in the follow-up of athletes lacks robust scientific evidence.

Events of sudden death in athletes constitute an important clinical scenario and the potential prevention of some situations through clinical cardiological evaluation raises the discussion about the need to use complementary methods in this evaluation. Although not all deaths in athletes are cardiovascular, pathologies such as hypertrophic cardiomyopathy and coronary anomalies are among the most frequent causes of sudden death during exertion in this population.^118,119^ Although the usefulness of anamnesis and physical examination is consensual, the need for TTE and even for the electrocardiogram in population screening of athletes is not a matter of general agreement among cardiology associations.^120^ However, if clinical evaluation suggests the likelihood of hypertrophic cardiomyopathy (or others of genetic origin), valvular heart disease or other structural cardiac changes, it becomes an essential investigative method (Table 9).

**Table 9:** Recommendations of transthoracic echocardiography in the evaluation of athletes of competitive and/or professional physical activities

Recommendation	Class of recommendation	Level of evidence
In the differentiation of "athlete's heart" from conditions of pathological hypertrophy	I	B
Assessment for release of competitive physical activity, when clinical consultation demonstrates the possibility of hypertrophic cardiomyopathy or other genetically transmissible ones	I	B
Assessment for release of competitive physical activity, when clinical consultation shows signs of valvular heart disease or other structural cardiac changes	I	C
In routine assessment of athletes when there is no suggestion of ventricular overload or hypertrophy by clinical consultation or ECG	IIb	C

ECG: electrocardiogram

## 3. Heart Murmurs, Valvular Heart Disease, Valvar Prostheses and Endocarditis

### 3.1. Heart Murmurs

Heart murmurs are common findings, with prevalence between 5 and 52%.^121^ They are produced when the laminar blood flow becomes turbulent, such as in stenoses or valve refluxes, emitting sound waves that can be detected with the aid of the stethoscope. It is important that during physical examination, even in asymptomatic patients, careful auscultation is performed in order to define its characteristics.^122^ An innocent murmur can be defined as a short, smooth ejective noise (1 to 2++ in 4), audible at the left sternal border, followed by a second normal sound, in the absence of other abnormalities.^123^ This finding, associated with normal chest x-ray and electrocardiogram, estimates a low probability of cardiac disease and, in this case, there is no need for complementary echocardiography.^124^ However, due to inadequate training or maintenance of knowledge, characteristics of the murmur or patient’s anatomy, the auscultation may leave doubts about the existence of underlying organic causes. In such situations, the use of electronic stethoscope^125^ and performing directed cardiac ultrasound,^126^ if available, may be useful. In case of persisting doubt or suspicion of cardiac alteration, the echocardiography should be performed (Table 10). This systematic approach and investment in medical training allows for a rational use of resources, avoiding excessive diagnosis and unnecessary exams.^124^

**Table 10:** Recommendations of transthoracic echocardiography in patients with murmur

Recommendation	Class of recommendation	Level of evidence
Asymptomatic patients, with murmur suggestive of cardiopathy	I	C
Asymptomatic patients with signs or exams (e.g., electrocardiogram) suggestive of cardiac disease	I	C
Patients with murmur and low probability of heart disease that cannot be ruled out by clinical investigation, electrocardiogram, chest X-ray or directed ultrasound	IIa	C
Patients without signs or symptoms suggestive of cardiopathy	III	C

### 3.2. Native Valves

Echocardiography is the standard diagnostic method for assessing heart valves. TTE should be performed in suspected and in diagnosed valvular heart disease, for evolutionary follow-up of moderate and important lesions, and changes in clinical status.^124^ The examination identifies the mechanisms involved, quantifies hemodynamic severity and repercussion, estimates prognosis, and assists treatment decision.^127,128^ In addition, physical exertion echocardiography can be performed to evaluate the behavior of echocardiographic parameters in asymptomatic patients and in cases of divergence between symptoms and the severity of the lesions estimated in the exams performed at rest.^24,127,129^ Besides traditional echocardiographic techniques, recent applications, such as the strain and the 3D, have provided new anatomical and functional information.^130-132^

#### 3.2.1. Mitral Regurgitation

TTE, in addition to diagnostic confirmation, provides information necessary for follow-up and decision making in mitral regurgitation(MR).^127^ TTE identifies dilation of cardiac cavities and dysfunction of both ventricles, in addition to allowing the classification of regurgitation into primary (due to valve lesions) or secondary (caused by changes in LV geometry). The 3D echocardiography is more accurate in volumetric measurements and left ventricular function; it can be useful in the evaluation of RV^4^ and allows better visualization of the valve apparatus and planning of interventions.^12,133^ The evaluation of the regurgitation degree can be made by the integrated approach of multiple qualitative and quantitative parameters: cavity dilations, pulmonary artery pressure, mitral inflow velocity, pulmonary vein flow pattern, mitral regurgitation density and duration analysis, calculus of the jet area or regurgitant volume, *vena contracta* measurement and regurgitant orifice measurement (flow convergence method, or proximal isovelocity surface area - PISA).^133^ Challenging situations for echocardiography are the presence of multiple and/or eccentric jets, cardiac arrhythmias and acute MR. In these cases, special emphasis should be given to integrated analysis relating anatomical and hemodynamic parameters. The improvement of quantitative measures, such as PISA and *vena contracta*, through the 3D echocardiography, can aid in the evaluation of eccentric reflux.^132,134^ Another important point is the measurement of left ventricular function, especially in asymptomatic patients, which can be overestimated by LVEF measurement, with implications for deciding the best time for intervention and for postoperative outcomes. Recently, measurement of myocardial deformation (strain) has been studied to more sensitively identify ventricular dysfunction, but despite good prospects, it still requires more studies and standardization.^131,135,136^

TEE, whether 2D or 3D, is indicated for evaluation of the regurgitation mechanism in the care of inappropriate transthoracic images or in discrepancies between echocardiographic and clinical parameters.^4,133^ The general recommendations for the use of the various modalities of echocardiography in MR are contained in tables 11, 12 and 13.

**Table 11:** Recommendations of transthoracic and stress echocardiography in mitral regurgitation

Recommendation	Class of recommendation	Level of evidence
Initial assessment of severity and MR mechanism	I	C
Periodic assessment of left ventricular dimensions and function in patients with moderate to severe MR without symptom changes	I	B
Patients with MR and modifications of signs or symptoms	I	B
Assessment in the first postoperative month	I	C
Assessment of hemodynamic changes and ventricular adaptation during pregnancy	I	C
Stress echocardiography in asymptomatic patients with severe MI to assess tolerance to physical efforts and hemodynamic changes	IIa	B
Stress echocardiography to assess discrepancy between severity of valve disease and symptoms	IIa	B
Stress echocardiography to evaluate left ventricular reserve	IIb	B
Assessment of ventricular mechanics (strain) for patients with borderline left ventricular function	IIb	B
3D TTE to assess preoperative anatomy and left ventricular function	IIb	C
Routine assessment of slight MR with LV normal function and dimensions	III	C

MR: mitral regurgitation; TTE: transthoracic echocardiography; LV: left ventricle

**Table 12:** Recommendations of transesophageal echocardiography in mitral regurgitation

Recommendation	Class of recommendation	Level of evidence
Intraoperative assessment to define the mechanism and to assist in valve repair	I	C
Unsatisfactory TTE for determination of severity and/or insufficiency mechanism, or for the assessment of LV function	I	B
Asymptomatic patients with severe MR to assess the possibility of valve repair	IIa	C
3D TEE to assess preoperative anatomy and left ventricular function	IIb	B
Assessment of patients with slight MR	III	C

TTE: transthoracic echocardiography; LV: left ventricle; MI: mitral regurgitation; TEE: transesophageal echocardiography

**Table 13:** Recommendations of echocardiography in patients with mitral valve prolapse

Recommendation	Class of recommendation	Level of evidence
Diagnosis, anatomical and functional evaluation of patients with physical signs of MVP	I	C
Confirmation of MVP in patients with previous diagnosis, but without clinical evidence to support it	I	C
Risk stratification in patients with clinic features or diagnosis of MVP	IIa	C
Exclusion of MVP in first-degree relatives of patients with myxomatous valve disease	IIb	C
Exclusion of MVP in patients with no suggestive physical signs or family history	III	C
Periodic echocardiographies in patients with MVP without insufficiency or with slight insufficiency, without alterations of symptoms or clinical signs	III	C

MVP: mitral valve prolapse.

#### 3.2.2. Mitral Stenosis

The diagnosis of mitral stenosis (MS) using echocardiography makes it possible to define its probable etiology as a consequence of a wide evaluation of the valve anatomy.^137^ The hemodynamic characterization of the gradients and valvular area, together with the description of thickening, leaflet mobility, subvalvar involvement and degree of calcification of the commissures, determines the progression stage of the disease and defines the most appropriate type of treatment when the disease is symptomatic. The joint interpretation of the echocardiography and the clinical symptoms determines the indication of surgical intervention or balloon catheter valvuloplasty.^137^ Recently, MS has been grouped into four distinct categories, based on anatomy, Doppler evaluation, presence of pulmonary hypertension, repercussions on LA, and symptoms: stage A (patients at risk for MS); stages B and C (asymptomatic patients, but with hemodynamic changes); and stage D (symptomatic patients with hemodynamic changes). Table 14 describes the parameters that must be included in the echocardiography to make this evaluation complete.^138,139^ The use of TTE usually defines the anatomy and severity of the lesion (Table 15), but there are indications for the use of TEE, such as in situations of technically difficult echocardiographic window or 24 hours before balloon catheter valvuloplasty to rule out the presence of thrombi in the LA (Table 16).^138,140,141^ 3D echocardiography, in the TTE or TEE modalities, has been shown to allow better anatomical analysis and more accuracy in the valvar area calculated by planimetry.^142,143^ Physical or pharmacological stress echocardiography (dobutamine) may be used in the discordance between symptoms and resting echocardiography data.^138^ Such phenomenon of incompatibility between symptoms and hemodynamic repercussion can result from the disproportion between the valve area and the patient’s body size, or the lack of complacency of the valve orifice (which should increase during exercise).^144^ On a low-dose dobutamine echocardiography, the mean mitral transvalvular gradient should increase above 18 mmHg^145^ in order for MS to be considered the cause of the symptoms, while on exercise echocardiography (treadmill), the significant cut-off value is one elevation above 15 mmHg.^138,144^ The increase in systolic pressure in the pulmonary artery is considered of clinical value only during the exercise echocardiography and should reach at least 60 mmHg so that pulmonary hypertension is considered secondary to MS. Other less frequent indications of a stress echocardiography may be found in asymptomatic patients with marked stenosis (Table 17).^144^ Care should be taken to diagnose associated lesions in MS, whether it is a significant MI (which imposes a limitation on balloon catheter valvuloplasty) or lesions on other heart valves.

**Table 14:** Elements of echocardiographic evaluation of mitral stenosis

Parameter	Description
Valve anatomy	Presence of dome, commissural fusion
Doppler	PHT value
Two-dimensional or three-dimensional	Planimetry of the mitral valve area
Left atrium	Indexed volume
Pulmonary artery pressure	Assessment of tricuspid or pulmonary insufficiency

PHT: pressure half-time.

**Table 15:** Recommendations of transthoracic echocardiography in mitral stenosis

Recommendation	Class of recommendation	Level of evidence
Establish diagnosis of patients with signs and symptoms of MS	I	B
Quantification of severity (PHT, gradients, valve area and pulmonary artery pressure)	I	B
Assessment of concomitant valve lesions	I	B
Determination of score for valvotomy by balloon catheter. Block Wilkins: thickening, mobility, subvalvar and calcification	I	B
Reassessment of stable MS with area < 1 cm^2^ each year Reassessment of stable MS with area between 1 and 1.5 cm^2^ every 2 years Reassessment of stable MS with area > 1.5 cm^2^ in 3 to 5 years Immediate reassessment with change of symptoms	I	B
Follow-up of balloon catheter valvuloplasty after dilatation	I	B
Assessment of hemodynamic alterations and adaptation during pregnancy	I	B

MS: mitral stenosis; PHT: pressure half-time

**Table 16:** Recommendations of transesophageal echocardiography in mitral stenosis

Recommendation	Class of recommendation	Level of evidence
Inconclusive transthoracic echocardiography	I	B
Assessment of thrombus preceding balloon catheter valvuloplasty	I	B
Assessment of the degree of mitral regurgitation preceding balloon catheter valvuloplasty (when there is doubt about transthoracic)	I	B

**Table 17:** Recommendations of stress echocardiography in mitral stenosis

Recommendation	Class of recommendation	Level of evidence
Discordance between symptoms and valvar area/gradient (mitral area > 1.5 cm^2^)	I	C
Assessment of asymptomatic patients with area < 1 cm^2^	IIa	C
Assessment of asymptomatic patients with an area between 1 and 1.5 cm^2^ in pregnancy or major surgery planning	IIb	C

#### 3.2.3. Aortic Stenosis

TTE is the first-line method (Table 18) for the diagnosis and assessment of the severity of aortic valve stenosis (AVS).^128,146-148^ The definition of the moment of surgical or percutaneous intervention depends on the integrated analysis of clinical and echocardiographic parameters (valvular anatomy, Doppler valvular hemodynamics and repercussion on cavity size and pulmonary artery pressure) that allow to classify aortic stenosis into four stages: stage A (risk of AVS); stage B (mild and moderate asymptomatic AVS); stage C (asymptomatic marked AVS), subdivided into C1 (with LVEF ≥ 50%) and C2 (LVEF < 50%); and stage D (classical symptomatic marked AVS).^149^ In some AVS subgroups, valve area is reduced in the low gradient and low flow periods, either due to the concomitance of left ventricular dysfunction (LVEF < 50%) or the presence of small and hypertrophied LV, despite preserved LVEF). These subgroups are designated as stage D2 (with decreased LVEF) or D3 (with normal LVEF).^146,147,150^ In these discrepancy situations, in which valve area is ≤ 1.0 cm^2^, the gradient is < 40 mmHg and LVEF is preserved (AVS with low paradoxical gradient or with low gradient and normal flow), additional methods such as TEE (3D, if possible), computed tomography or cardiac resonance may be necessary to confirm the severity of AVS.^147^ TEE allows a better evaluation of the aortic valve anatomy (valvular calcification), etiology (degenerative, congenital or rheumatic), LV exit path (diameter and geometry, mainly 3D) and greater accuracy in the calculation of valvular area, either by continuity equation or by direct planimetry.^147,150^ The absence of significant calcification should alert to the possibility of sub- or supravalvar obstruction. In good-quality images, 3D TTE also allows for a more accurate LVEF assessment and a calculation of ejection volume (aortic transvalvular flow) by subtraction of final diastolic and systolic volumes, without the need to use LV exit pathway measurement and Doppler. This calculation, however, should be analyzed along with the other parameters, once it may also underestimate aortic transvalvular flow.^147,150^ If the calculation of the valve area is made during hypertension (blood pressure ≥ 140 x 90 mmHg), it should be repeated after blood pressure control because it may underestimate the transvalvar flow. Reduction in LV systolic function by GLS measurement, with no other explanation, in the presence of preserved LVEF, favors the diagnosis of severe AVS with low paradoxical gradient. Stress echocardiography with low dose of dobutamine, with calculation of the projected area of ​​the valve, if necessary,^144,147^ should be performed if there is marked aortic stenosis with low-flow/low gradient and left ventricular dysfunction (stage D2) to distinguish truly marked stenosis from pseudostenosis and to evaluate the contractile reserve (Table 19). Echocardiography under physical stress is recommended to unmask symptoms or to provide prognostic information on moderate or marked asymptomatic AVS with preserved LVEF (stages B or C) (Table 19). An increase in the mean pressure gradient (> 18 to 20 mmHg), the absence of contractile reserve and the increase of pulmonary artery systolic pressure (PASP) > 60 mmHg during exercise are parameters of worse prognosis and require follow-up at shorter intervals.^144^ Stress echocardiography may also be useful in low-flow/low paradoxical gradient (with preserved LVEF), asymptomatic or with mild or doubtful symptoms, to confirm the severity of AVS using the same criteria.^144^ In suboptimal images, the evaluation of LVEF can be improved by the use of myocardial contrast, to better delineate endocardial borders.^147^ The invasive hemodynamic study is restricted to situations in which noninvasive imaging tests are inconclusive.^146,149^ The follow-up interval with TTE depends on the stage of the disease and predictors of poor prognosis. Accentuated aortic valve calcification is another predictor of more severe stenosis and worse clinical progression.^151^ Marked AVS with low paradoxal gradient has worse prognosis when compared to classical AVS and the remaining AVS subgroups.^150^ The paradoxical low gradient and normal flow (LV > 35 mL/m^2^) seems to have a prognosis similar to that of moderate AVS, but should be accompanied by shorter intervals, particularly if symptomatic.^147^ In classical AVS, the maximum velocity ≥ 5m/s^152^ and an annual increase in maximum velocity ≥ 0.3cm/s^151^ in serial examinations (recorded in the same incidence and with the same quality) are predictors of worse prognosis and of faster progression.^146,147,149^

**Table 18:** Recommendations of transthoracic echocardiography in aortic valve stenosis

Recommendation	Class of recommendation	Level of evidence
Diagnosis and assessment of the severity of AVS in the presence of suspicious murmur or symptoms potentially related to AVS, such as: chest pain, dyspnea, palpitations, syncope, stroke or peripheral embolic event	I	B
Syncope	I	B
History of bicuspid AV in first degree relatives	I	B
Patients with AVS to assess wall thickness, LV size and function	I	B
Reassessment of patients with the AVS diagnosis with change of symptoms or signs	I	B
Suboptimum transthoracic contrast echocardiography (≥ 4 contiguous LV segments not seen), for assessment of LV function and calculation of the ejected volume	I	B
Annual reassessment of asymptomatic patients with marked AVS (maximal velocity ≥ 4 m/s) (stage C1), with reduction of the interval to 6 months if there are predictors of greater severity at rest (AV marked calcification, maximum velocity > 5.5 m/s, increase in maximal velocity ≥ 0.3 m/s/year and low-flow/low paradoxical gradient) or effort echocardiography*	I	B
Reassessment every 1 to 2 years of asymptomatic patients with moderate AVS (maximal velocity 3 to 3.9 m/s) (stage B), with reduction of the interval to 1 year if there are predictors of greater severity on echocardiography at rest or effort echocardiography*	I	B
Reassessment of asymptomatic patients with discrete AVS (maximal velocity 2 to 2.9 m/s) (stage B), every 3 to 5 years, with reduction for 1 year in the presence of marked calcification	I	B
Reassessment after hypertension control in patients with accentuated AVS with low-flow/low gradient and preserved LVEF	I	B
Monitoring of percutaneous implantation of aortic valve prostheses and results immediately after implantation (catheter, position, prosthesis function, regurgitation)	I	B
Assessment of complications immediately after percutaneous implantation of the aortic prosthesis (hypotension, coronary occlusion, LV dysfunction, LVOT obstruction, marked mitral insufficiency, prosthesis displacement, tamponade, right ventricular perforation, gas embolism, aortic dissection)	I	B
Early assessment (within 30 days) after percutaneous implantation of aortic prosthesis as to the degree of valve regurgitation (or paravalvarization) in the presence of suspected valve dysfunction	I	B
Reassessment in less than one year of changes in hemodynamic severity and LV function in patients diagnosed with moderate AVS, before or during pregnancy, or who will be submitted to situations of increased demand (non-cardiac surgery)	IIa	C
Good quality transthoracic 3D echocardiography for better assessment of valve morphology (especially in suspected bicuspid AV) and the degree of calcification	IIb	B
3D echocardiography in good-quality transthoracic image in symptomatic acute AVS with low gradient and preserved LVEF (D3), to reassess the diameter and geometry of the LVOT, to calculate the valvular area by planimetry or to calculate the valvular area by the continuity equation using the ejected volume measured directly by 3D (instead of the ejected volume derived from Doppler or two-dimensional Simpson)	IIb	B

AVS: aortic valve stenosis; AV: aortic valve; LV: left ventricle; LVEF: left ventricle ejection fraction; LVOT: left ventricular outflow tract; 3D: three-dimensional.

* Predictors of worse prognosis on resting echocardiography: marked aortic valve calcification and maximal velocity increase ≥ 0.3 m/s/year; on exercise echocardiography: increased mean pressure gradient (> 18 to 20 mmHg), absence of contractile reserve, and increased PASP (> 60 mmHg).

**Table 19:** Recommendations of stress echocardiography in aortic valve stenosis

Recommendation	Class of recommendation	Level of evidence
Low-dose dobutamine stress echocardiography to confirm symptomatic low-flow/low gradient AVS and reduced LVEF and to assess the presence of contractile reserve (stage D2)	I	B
Stress echocardiography in asymptomatic patients with moderate or marked AVS (stages B and C1) to assess exercise-induced symptoms, abnormal responses to systemic or pulmonary arterial pressure, and behavior of gradients and left ventricular function	IIa	B
Stress echocardiography in asymptomatic patients (or with mild or doubtful symptoms) with low-flow/low gradient AVS and preserved LVEF to differentiate true stenosis from aortic pseudostenosis	IIb	B
Stress or dobutamine echocardiography in symptomatic marked AVS	III	C

AVS: aortic valve stenosis; LVEF: left ventricle ejection fraction

In patients who are candidates for percutaneous implantation of aortic prosthesis for the treatment of AVS, 3D TEE can be used to evaluate the diameter of the ring, but it depends on the operator and image quality and should be used only when there is contraindication to computed tomography. On the other hand, 3D TEE is recommended to monitor the procedure and to evaluate outcomes or complications (Table 20).^148^

**Table 20:** Recommendations of transesophageal echocardiography in aortic valve stenosis*

Recommendation	Class of recommendation	Level of evidence
High acuity AVS with low-flow/low gradient and preserved LVEF (D3), for assessment of valve area (reassessment of LVOT measurement) and assessment of valve morphology, including degree of calcification	I	B
Acute AVS with low flow/low gradient and reduced LVEF (D2) for assessment of valve morphology, including degree of calcification	IIb	B
Disagreement between the severity of AVS and transthoracic examination and clinical evaluation	I	B
Difficulty assessing AVS at transthoracic examination due to inadequate acoustic window	I	B
Assessment of aortic valve annulus size and geometry in patients candidates for percutaneous aortic valve prosthesis implantation	I	B
Monitoring of percutaneous implantation of aortic valve prostheses and results immediately after implantation (catheter, position, prosthesis function, regurgitation)	I	B
Assessment of complications immediately after percutaneous implantation of the aortic prosthesis (hypotension, coronary occlusion, LV dysfunction, LVOT obstruction, marked mitral insufficiency, prosthesis displacement, tamponade, right ventricular perforation, gas embolism, aortic dissection)	I	B
Early assessment (within 30 days) after percutaneous implantation of aortic prosthesis as to the degree of valvular or paravalvular regurgitation ) in the presence of suspected valve dysfunction	I	B
Stroke after percutaneous implantation of aortic prosthesis in case of suspected valve dysfunction	I	B
Assessment of the distance of the aortic valve annulus to the coronary sinus in patients candidates for percutaneous implantation of aortic valve prosthesis	IIb	B

* 3D, if available. AVS: aortic valve stenosis; LVEF: left ventricular ejection fraction; LVOT: left ventricular outflow tract; LV: left ventricle

#### 3.2.4. Aortic Regurgitation

Echocardiography is the first-choice method to confirm diagnosis and etiology and to assess the severity and hemodynamic consequences of aortic regurgitation (AR).^133,153^ AR can be observed due to primary diseases of the aortic valve (AV) or to abnormalities of the aortic root and ascending aorta. Degeneration of AV and bicuspid aortic valve are the most common etiologies. Other causes include rheumatic fever, fibrosis or infection, alteration of the valvular apparatus support or dilatation of the valve ring. The integrated analysis of clinical and echocardiographic parameters (valve anatomy, aortic root and ascending aortic root diameters, Doppler valvular hemodynamics and repercussions on cavity size and pulmonary artery pressure) allows the classification of AR in four stages: stage A (risk of AR), stage B (mild to moderate asymptomatic AR), stage C [asymptomatic acute AR without (C1) or with dysfunction/dilation of LV (C2)]; and stage D (symptomatic acute AR).^149^ In suboptimal images, LVEF measurement may be more accurate with the use of myocardial contrast to delineate the endocardial borders.^133^ TEE (with 3D if available), tomography or cardiac resonance may be necessary to better assess the aortic root and ascending aorta (especially in the case of a bicuspid aortic valve), the severity of AR, or the quantification of LV ejection volumes and ejection fraction.^133^ The appearance of symptoms in AR drastically changes prognosis. Effortless echocardiography may be indicated to reveal the presence of symptoms or to investigate other causes not related to AR (diastolic dysfunction, pulmonary hypertension or dynamic MI).^144^ However, it should not be used to assess severity, once that increased heart rate shortens diastole, limiting quantification.^144^ The follow-up interval with TTE depends on the stage of the disease and the presence of aortic dilatation associated with bicuspid aortic valvopathy.^148,154^ Recommendations for the use of the various modalities of echocardiography in AR are set out in tables 21 to 24.

**Table 21:** Recommendations of transthoracic echocardiography in aortic regurgitation^153^

Recommendation	Class of recommendation	Level of evidence
Confirm the presence, etiology, and severity of acute or chronic AR	I	B
In patients with dilation of the aortic root to assess the degree of AR and the magnitude of aortic dilatation	I	B
Reassessment of patients with prior AR and change of symptoms or signs	I	B
Annual reassessment of LV size and function in marked asymptomatic AR, with reduction of the interval to six months for the first examination, or if there are significant changes in diameters or LVEF on subsequent examination (stage C)	I	B
Reassessment every one to two years in moderate asymptomatic AR (stage B)	I	C
Reassessment every three to five years in asymptomatic mild AR (stage B)	I	C
Reassessment in less than one year of hemodynamic severity and LV function in patients diagnosed with AR before or during pregnancy, or who will undergo situations that increase demand (non-cardiac surgery)	IIa	C

AR: aortic regurgitation; LV: left ventricle; LVEF: left ventricle ejection fraction

**Table 24:** Recommendations of transesophageal echocardiography in aortic regurgitation* ^153^

Recommendation	Class of recommendation	Level of evidence
Discrepancy between qualitative and quantitative parameters of transthoracic echocardiography and/or between echocardiography and clinical evaluation regarding the severity of AR	I	B
Confirm the presence, etiology and severity of acute AR if the transthoracic echocardiography is of limited, doubtful or inconclusive quality	I	B
In patients with a bicuspid aortic valve to assess the diameter of the aortic root and ascending aorta when the transthoracic image is suboptimal	I	B

* 3D, if available. AR: aortic regurgitation

#### 3.2.5. Tricuspid Valvulopathy

TTE is the first-line method for evaluating tricuspid valve abnormalities (Table 25).^146,148,149^ In most cases, tricuspid regurgitation (TR) is secondary to tricuspid ring dilatation and leaflet pull due to distortion and right ventricular remodeling, which occur due to volume or pressure overload caused by diseases of the left side of the heart , pulmonary hypertension, pulmonary valve stenosis, among others. In this context, leaflets are structurally normal. Primary causes of TR are rarer and may be due to infective endocarditis (mainly drug users), rheumatic heart disease, carcinoid syndrome, myxomatous disease, endomyocardial fibrosis, corneal rupture related to endomyocardial biopsy, Ebstein’s anomaly, and congenital dysplasia, among others.^133^ Similar to mitral and aortic valve disease, it can be classified into four stages (A to D).^149^ A thorough analysis of valvular anatomy by TTE is fundamental for the diagnosis of the etiology and mechanisms involved. It is necessary to measure the diameter of the ring and the use of all indexes of RV systolic size and function.^133^ These measures help in decision making regarding the moment of the surgery and in the surgical planning. In situations of doubt regarding the RV, 3D TTE can be used, although it still requires more validation. Cardiac resonance remains the gold standard.^146^ In this context, TEE is not recommended due to the anterior location of the RV, which makes it difficult to see through the transesophageal route.148 Significant primary TR requires intervention before RV impairment.^146,149^ Secondary TR is usually treated when left side valve disease is corrected. As in the other valvopathies, the echocardiographic follow-up interval depends on the stage of the disease, but the etiology of the disease must also be considered. In the case of secondary TR, it is appropriate to follow the recommendations described for left heart valve dysfunctions. Significant annular dilatation (≥ 40 or > 21 mm/m^2^) and dilatation or progressive RV dysfunction should alert for earlier follow-up.^133^ Tricuspid stenosis (TS) is an uncommon condition that, if present, is frequently associated with TR of rheumatic origin.^146,149^ In this case, the presence of associated mitral stenosis is common, which is usually the predominant lesion. Other causes are rare, such as congenital diseases, drugs, Whipple’s disease, endocarditis, and large right atrial tumor.^146^ TS diagnosis of is often neglected. Careful analysis of the subvalvular apparatus is essential to predict valve repair.^146^ The integration of clinical and echocardiographic parameters related to TS (mean gradient > 5 to 10 mmHg, valve area ≤ 1.0 cm^2^ and mean time of pressure drop ≥ 190 ms) classifies severity in stages C (marked asymptomatic) and D (marked symptomatic).^149^

**Table 25:** Recommendations of the echocardiography in tricuspid valvulopathy

Recommendation	Class of recommendation	Level of evidence
2D TTE is recommended to confirm diagnosis, to assist in identifying the etiology and mechanisms of tricuspid lesions, to determine severity, to assess pulmonary pressure, as well as the dimensions of the cardiac cavities and the right ventricle function and to characterize any associated cardiac disease on the left side	I	B
TEE (with 3D if available) may be used for more detailed assessment of valve morphology, mechanisms and Doppler quantification if the TTE is of limited, doubtful or inconclusive quality, or there is a discrepancy between the clinical data and the echocardiographic findings	I	B
3D TTE (in optimal windows) can be used to assess systolic and diastolic volumes and RV systolic function in patients with marked TR (stages C and D)	IIb	B
TEE (2D or 3D) for assessing the systolic function of the RV in marked TR	III	C

TTE: transthoracic echocardiography; 2D: two dimensional; TEE: transesophageal echocardiography; 3D: three dimensional; RV: right ventricle; TR: tricuspid regurgitation

#### 3.2.6. Pulmonary Valvulopathy

TTE is the initial recommended method to diagnose and evaluate the severity of pulmonary stenosis (PS) or regurgitation (PR), its etiology and effects on cardiac structure and right ventricular function (class I).^133,149^ In addition to assessing the valvular anatomy, investigation of the etiology requires a thorough evaluation of the RV, pulmonary ring, pulmonary artery trunk and its branches. Primary PS or PR (with leaflet involvement) are more often due to congenital diseases than acquired ones. Secondary PR occurs in situations of pulmonary hypertension. There is little literature on the quantification of the severity of PR on the echocardiography, but there is a consensus that it should be done in an integrated way with pulsed, continuous and color Doppler parameters; and graded as mild, moderate, or marked.^133^ PS and PR are classified, from the clinical-echocardiographic point of view, as stage C (marked asymptomatic) and D (marked symptomatic).^149^ Evaluation of the pulmonary valve may be difficult for TTE. In this situation, however, the TEE does not provide additional information and is not recommended (class III). There is little data on the value of 3D echocardiography. In cases of limited transthoracic imaging or severity parameters inconclusive or discordant with clinical data, cardiac resonance is recommended as the best method.^133^

#### 3.2.7. Associated Valvar Lesions

Associated valvular lesions (AVL) in our setting are frequent due to the high prevalence of rheumatic fever (RF), which reaches 70% of valvular heart disease in Brazil.^141^ In the EuroHeart Survey, 51% of AVL patients had RF and 40% had degenerative valve disease.^155^ Pathophysiology is complex, because it depends on the specific combination of each valve lesion, and its diagnosis is challenging because the guidelines provide us with specific valvular parameters alone. AVL may result from two primary valvular diseases^156^ or from the combination of primary and secondary valvular disease.^157^ Despite the high prevalence of AVL, there is little evidence of the best course of action to be taken in each combination. The most common combinations and their most frequent changes are reported below:^156^

- AS and MI: Increased LV pressure caused by aortic stenosis may increase the regurgitant orifice and decrease aortic transvalvular gradients, mimicking a low-flow state.^156,158^ In some AS cases, there may be MR secondary to dilation and left ventricular dysfunction (tethering). Less frequent, but possible in these patients, is the presence of primary MR.
- AS and MS: are cases of difficult clinical control, in which the patient rapidly evolves to low throughput states. The gradients of both valves may be underestimated, and if the patient is inadvertently submitted to balloon catheter valvotomy of the mitral valve, acute pulmonary edema may occur due to the lack of LV compliance as a consequence of AS.^141,156^
- AR and MS: the presence of MS limits the increase in ventricular volumes frequently observed in AR; which may underestimate the severity of AR.^141,156^
- MR and AR: as a consequence of the volume overload imposed by both valvopathies, these patients usually have earlier contractile deficit than with each isolated valvopathy and progress more rapidly to the symptomatic phase of the disease.^141,156,158^

Recommendations for TTE and TEE in AVL are listed in tables 26 and 27, respectively. The frequency at which TTE should be performed is debatable and depends on the type of AVL and symptomatology; in general, the examination should be repeated according to the predominant valve lesion guideline.^156^ In the case of balanced lesions, TTE should be repeated with a shorter interval than the one suggested for a single valve lesion.^156^

**Table 26:** Recommendations of transthoracic echocardiography in associated valve lesions

Recommendation	Class of recommendation	Level of evidence
Establish diagnosis of patients with multiple murmurs	I	C
Quantification of the severity of stenoses and associated insufficiencies	I	C
Immediate reassessment with change of symptoms	I	C
Annual reassessment of asymptomatic patients with AVL	IIa	C

AVL: associated valvular lesions

**Table 27:** Recommendations of transesophageal echocardiography in associated valve lesions

Recommendation	Class of recommendation	Level of evidence
Inconclusive transthoracic echocardiography	I	C
Doubts in the quantification of valvular lesions	I	C
Monitoring of invasive procedures for injuries that can be treated percutaneously	I	C

### 3.3. Valvular Prostheses

TTE is recommended as a first line examination for the analysis of valvular prostheses. TEE may be necessary when it is necessary to better evaluate the structure and complications of valvular prostheses, recommended in cases of dysfunction (Table 28). When performing the echocardiographic examination of valvular prostheses, it is necessary to know and document the reason for the investigation, the patient’s symptomatology, the type and size of the prosthesis, the date of surgery, blood pressure, heart rate, height, weight and the patient’s body surface area.24 A detailed postoperative TTE is recommended four to six weeks after surgery, when the thoracic surgical incision is healed, thoracic wall edema resolved, and left ventricular function recovered. In this examination, it is important to record: cavitary dimensions, ventricular function, prosthetic gradients, valvular areas, presence of functional or pathological refluxes, pulmonary pressure and alterations of other valves; defining the basal conditions of valve prostheses, since the examination will be taken as reference for serial assessments. Regarding the periodicity of TTE in patients with prosthesis, a frequent assessment in asymptomatic patients with supposedly normal mechanical prosthesis is not recommended. For biologic prostheses considered to be normal, exams after five (ESC)^159^ or ten years (ACC/AHA) are recommended).^127^ However, annual examinations are recommended in patients with new design prostheses that have not had their proven durability in patients with aortic dilatation at the time of surgery and in patients with mitral prostheses to evaluate the evolution of tricuspid regurgitation and RV function. Echocardiographic investigation (TTE and TEE) is recommended when changes in cardiac auscultation, onset of symptoms or suspicion of prosthesis dysfunction occur. In cases where there is clinical suspicion of infective endocarditis or thrombosis, the analysis should be more thorough.^123^ In cases of significant reflux of prostheses, it is recommended to perform evolutionary TTEs every three to six months.^128^ TEE, due to its proximity and posterior approach to the heart, achieves better diagnostic precision in valvular prosthesis dysfunctions. In fact, TTE and TEE complement each other, since the TTE better evaluates the changes in flow and TEE, the morphological changes. It is always advisable to carry out the full and careful TTE before recommending the TEE. The 3D TEE^160^ provides additional information about the 2D image, particularly regarding the spatial relationship of the structures around the prosthesis, the direction and extent of regurgitant jets, the location of paravalvular leaking and the identification, position and number of larger anomalous prosthetic or periprosthetic echoes, potentially more embolinogenic.^161^ The diagnosis of prosthesis stenosis should always be performed with the extensive use of echocardiography. The transprosthetic gradients are variable in each model and size, and there may be high gradients in cases of small, even normal, prostheses when implanted in large body surface patients, a finding known as mismatch.^162^ Patients who remain with significant LVH in the late postoperative period may also present elevated gradients after aortic prosthesis implantation. Thus, the comparison with the basal echocardiography is always important. In biological prostheses, the most frequent cause of stenosis is the degeneration and calcification of the leaflets, usually a late complication. In mechanical prostheses, the growth of fibrous tissue into the ring, known as pannus, is also a late complication that can cause stenosis, reflux, or double prosthetic dysfunction. The detection and quantification of the reflux of the prostheses are usually hampered by the acoustic shadow caused by the mechanical prostheses, mainly in the mitral position. In such cases, TEE can aid in the detection and quantification, and determine if the insufficiency is prosthetic or periprosthetic, functional or pathological. We must be careful in differentiating the “physiological” refluxes, which are common in prostheses, from the pathological ones.^163^ In general, physiological reflux presents laminar flow at color Doppler and pathological reflux presents a turbulent, color mosaic flow. In cases of suspicion of infective endocarditis in prosthetics, the diagnosis is made difficult by the presence of shadows and reverberations, allowing the TTE to identify only the large vegetation. Given the clinical suspicion of endocarditis, it is always advisable to perform the TEE, which has greater sensitivity, detecting smaller vegetations and possible complications, such as annular abscesses (Table 29). 3D TEE allows a more precise spatial localization, in relation to the adjacent prosthetic and anatomical structures, of potentially emboligenic vegetations. In cases of embolic phenomena or acute stenosis of the prosthesis, especially in the mitral position, the presence of valve thrombosis or strands (fibrin) should be suspected, with TTE and TEE being indicated (3D TEE, if possible) in order to overcome the acoustic shadow and better observe the LA and the atrial face of the prosthesis. In these cases, in addition to looking for thrombi or fibrin in the valve or the LA, the prosthesis’ mobile structures and the emboligenic potential of the thrombi should be functionally evaluated (Table 30).

**Table 28:** Recommendations of echocardiography in valvular prostheses

Recommendation	Class of recommendation	Level of evidence
TTE in patients with valve prostheses with changes in clinical signs or symptoms, suggesting prosthetic dysfunction (stenosis or insufficiency)	I	A
TEE in patients with prosthetic dysfunction to TTE, for confirmation and better quantification of dysfunction	IIa	B
Periodic reassessment in patients with prosthesis, with ventricular dysfunction without modification of symptoms or clinical signs	IIa	B
Periodic reassessment in biological valve prostheses without signs or symptoms of prosthetic dysfunction	IIb	B

TTE: transthoracic echocardiography; TEE: transesophageal echocardiography

**Table 29:** Recommendations of echocardiography in infective endocarditis in patients with valve prostheses

Recommendation	Class of recommendation	Level of evidence
Detection and characterization of the valve lesion and evaluation of the hemodynamic repercussion*	I	B
Detection of complications such as abscesses, ruptures and fistulas*	I	B
Reevaluation in cases with poor clinical evolution*	I	B
Suspected endocarditis in patient with negative cultures*	I	B
Bacteremia of unknown etiology*	I	B
Persistent fever with no evidence of bacteremia or new murmurs*	IIa	B
Routine assessment during treatment of uncomplicated endocarditis*	IIb	B
Transient fever without evidence of bacteremia or new murmur*	III	B

* Transesophageal echocardiography may give additional information to those obtained with transthoracic echocardiography

**Table 30:** Recommendations of echocardiography in patients with clinical suspicion of valvular prosthesis thrombosis

Recommendation	Class of recommendation	Level of evidence
Carrier of mechanical prosthesis with embolic phenomenon and/or acute heart failure	I	B
Assessment to determine the hemodynamic changes caused by thrombosis	IIb	B
TEE to complement the TTE, to evaluate the mobility and emboligenic potential of the thrombi and functional study of the prosthesis	IIb	B

TEE: transesophageal echocardiography; TTE: transthoracic echocardiography

### 3.4. Infective Endocarditis

Infective endocarditis (IE) is vascular or cardiac endocardial infection resulting from invasion of microorganisms. Despite the advances in diagnostic techniques and treatment, mortality by IE remains high.^164^ The profile of the disease presentation changed, with the emergence of new risk groups and more virulent microorganisms, with staphylococci emerging as the main etiological agents. Echocardiography is fundamental in the IE approach (Table 31).^165-168^ The best resolution of the devices and, especially, the use of TEE are responsible for the high accuracy of the method in the diagnosis and evaluation of complications. The additional value of TEE when TTE is not diagnostic is well defined in the strong clinical suspicion of IE and/or the presence of valvular prostheses. However, the indication of TEE as an initial examination needs to be validated by new studies.^168^ The definitive diagnosis of IE is based on positive blood cultures and/or characteristic echocardiographic findings. The findings following the echocardiography are major diagnosis criteria: vegetation defined by a mobile condensed mass, adhered to the valvar endocardium, mural or implanted prosthetic material; abscesses or fistulas; new prosthesis dehiscence (especially when it occurs late after its implantation) and new valve regurgitation.^169^

**Table 31:** Recommendations of transthoracic echocardiography and transesophageal echocardiography in infectious endocarditis

Recommendation	Class of recommendation	Level of evidence
TTE is indicated as the first examination in the clinical suspicion of IE	I	B
TEE is indicated on clinical suspicion of IE and negative or non-diagnostic TTE	I	B
TEE indicated in the diagnostic suspicion of IE in patients with valvular prostheses and intracardiac devices	I	B
Indicated to repeat TTE or TEE within five to seven days in the face of high clinical suspicion and initial negative TEE	I	C
Echocardiography indicated for the assessment of staphylococcal bacteremia of unknown source	IIa	B
TEE may be indicated for suspected IE, even in cases with positive TTE with good quality and reliable findings (except isolated IE)	IIa	C
New TTE or TEE indicated for suspected new complications (abscesses, perforations, embolisms, persistence of fever, heart failure)	I	B
New TTE or TEE indicated for the follow-up of uncomplicated IE, for vegetation size monitoring or detection of silent complications. The type (TEE or TTE) and the date of the new examination will depend on the initial findings, type of microorganism and response to therapy	IIa	B
Intraoperative TEE in all cases of valve surgery by IE	I	B
At the end of the treatment to establish new parameters of cardiac and valvular morphology and function	I	C

TTE: transthoracic echocardiography; IE: infective endocarditis; TEE: transesophageal echocardiography

## 4. Hypertension and Pulmonary Thromboembolism

Pulmonary hypertension (PH) is a clinical condition associated with high morbidity and mortality, the prevalence of which is unknown due to different presentation groups. From the knowledge of the various pathophysiological mechanisms, the current classification divides PH into five groups.^170^ Regardless of the mechanism, it is defined as mean pulmonary arterial pressure greater than or equal to 25 mmHg, at rest, documented by cardiac catheterization.^170^ Currently, TTE is considered a method of fundamental importance in the initial evaluation of patients with clinical suspicion of PH (Table 32), since it offers information related to: diagnosis, hemodynamic status, therapeutic response and prognosis.^171^ Hemodynamic data, such as pulmonary artery systolic pressure, mean arterial pressure, pulmonary artery occlusion pressure and blood volume (assessed by varying the size of the inferior vena cava), can be measured by this method.^172^ The presence of RV hypertrophy, enlargement of the right cavities, anomalous movement of the septum and pericardial effusion suggest the diagnosis. The analysis of the contractile function of the RV is performed through the TAPSE, s-wave (systolic) of the tissue Doppler, RV area fractional variation and ejection fraction to the 3D.^4^ Right cardiac catheterization remains the gold standard for diagnosis, since it allows the direct measurement of hemodynamic data in the pulmonary circulation and evaluates the capacity of response to vasodilator therapy through the pulmonary vasoreactivity test.

**Table 32:** Recommendations of the echocardiography in pulmonary hypertension and thromboembolism^170,174^

Recommendation	Class of recommendation	Level of evidence
TTE recommended as a first line examination for noninvasive diagnostic investigation of suspected pulmonary hypertension	I	C
TTE recommended in the assessment of signs of pulmonary hypertension in symptomatic patients with portal hypertension or liver disease and in all indicated to hepatic transplantation	I	B
TTE recommended as an initial examination for the assessment of pulmonary hypertension in patients with systemic sclerosis and annually	I	C
TTE recommended for noninvasive diagnostic assessment of patients with pulmonary disease with suspected pulmonary hypertension	I	C
High-risk pulmonary embolism, in the presence of shock or hypotension, TTE at the bedside or angiotomography (depending on the patient's clinical conditions or availability)	I	C
High-risk pulmonary embolism with signs of right ventricular dysfunction, unstable for angiography (TTE at the bedside with Doppler of lower limbs and/or TEE to assess pulmonary artery thrombus)	IIb	C
Not recommended in asymptomatic HIV positive patients for the detection of pulmonary hypertension	III	C

TTE: transthoracic echocardiography; TEE: transesophageal echocardiography; HIV: human immunodeficiency virus

Pulmonary thromboembolism (PTE) is another clinical condition of high mortality, which can cause complications such as chronic thromboembolic pulmonary hypertension (PH group IV).^170^ Clinical suspicion, progress in diagnosis, and effective therapy are critical in reducing mortality in the acute event. The sensitivity and specificity of TTE for the diagnosis of PTE are 50 to 60% and 80 to 90%, respectively. In critical patients, TEE may increase this sensitivity. The visualization of the thrombus in the right atrium (RA), in the RV or in the trunk of the pulmonary artery ratifies the diagnosis. However, indirect signs are more commonly found, such as dilatation of the right cavities, RV contractile dysfunction, interventricular septal flattening, McConnel’s signal (apical region with preserved contractility and akinetic mean free wall segment, with sensitivity of 77% and specificity of 94%) and dilation of the inferior vena cava. The pulmonary artery acceleration time is a parameter with good sensitivity, since it is altered (< 100 ms) in cases of small pulmonary embolism.173 RV strain is an important tool because it shows the segment that presents reduced value and evaluates its deformity after reperfusion therapy. Patients who develop contractile dysfunction of the RV or patent foramen ovale present a reserved prognosis.

## 5. Coronary Artery Disease

### 5.1. Introduction

Coronary artery disease has a wide clinical spectrum, ranging from asymptomatic severe disease,^175^ long-term stable angina or acute coronary syndrome (ACS)/acute myocardial infarction (AMI) with hemodynamic instability as the first manifestation of the disease.^176,177^ The echocardiography has applications in its diagnostic recognition, stratification of risk in the acute phase, follow-up and determination prognosis in the long term.^178,179^

### 5.2. Acute Coronary Syndrome

#### 5.2.1. Transthoracic Echocardiography

In the scenario of a patient with acute chest pain and suspected coronary artery disease, echocardiography may be useful (Table 33) and should be routinely available in the emergency department and thoracic pain units.^180^ Evidence of new or presumably contractile change from LV to TTE is one of the parameters in the third universal definition of myocardial infarction^181^ and may in fact assist in the diagnosis/prognostic determination of an ACS. In addition, TTE may aid in the differential diagnosis of chest pain and/or associated conditions, such as acute aortic dissection, aortic stenosis, hypertrophic cardiomyopathy, and pulmonary embolism.^180^ By dividing LV into 16 or 17 segments, contractile segmental function is visually quantified based on systolic thickening (ST): hyperkinetic = 0 (ST > 70%); normal = 1 (ST = 50 to 70%); hypokinetic = 2 (ST < 40%); akinetic = 3 (ST < 10%); “dyskinetic = 4 (paradoxical movement/systolic expansion)”. The wall motion score index (WMSI) is the reference parameter to express the LV segmental function and its normality value is 1; values between 1 and 1.6 show a contractile alteration of mild degree; while WMSI values above 1.6 indicate greater involvement and worse prognosis.^182^ Obviously, the absence of alterations in segmental contractility in resting TTE does not exclude the presence of coronary artery disease.^180^ It should also be considered that the contractile alteration may occur in other conditions such as myocarditis, RV overload (volume/pressure), ventricular pre-excitation, Takotsubo type cardiomyopathy, left bundle branch block, chagasic cardiomyopathy or presence of pacemaker.^180^ TTE is the exam of choice in cases of hemodynamic instability with suspected cardiac origin, as well as in the identification of mechanical complications of AMI.^179,180^ However, it is necessary to avoid requesting the examination for evaluation of patients with chest pain with a confirmed diagnosis of myocardial ischemia (ACS/AMI), since TTE should not delay the immediate onset of treatment.^176,177,180^ On the other hand, in the screening of symptomatic patients suspected of having coronary artery disease in the emergency room, recent evidence indicates the potential usefulness of GLS calculated by 2D speckle tracking. In the absence of preexisting structural heart disease, previous infarction or left bundle branch block, GLS (when < 16.5%) may complement existing diagnostic algorithms and act as an early adjunct marker of ischemia.^183^

**Table 33:** Recommendations of transthoracic echocardiography in acute coronary syndrome

Recommendation	Class of recommendation	Level of evidence
Assessment of global and segmental ventricular function	I	C
Differential diagnosis of alternative causes of chest pain: severe aortic stenosis, hypertrophic cardiomyopathy, pulmonary embolism, aortic dissection*, pericarditis and the presence of cardiac tumors	I	C
Chest pain with hemodynamic instability and suspected cardiac origin	I	C
Suspected mechanical complications in myocardial infarction: left ventricular aneurysm, rupture of free wall or papillary muscle, ventricular septal defect, pericardial effusion	I	C
Assessment of right ventricular impairment in the presence of inferior wall myocardial infarction	I	B
During chest pain of possible ischemic origin, with electrocardiogram and non-conclusive cardiac enzymes	IIa	B
Calculation of global longitudinal strain using speckle tracking as an adjunct to existing diagnostic algorithms and risk classification in patients with suspected coronary disease&	IIa	B
Assessment of patients in the presence of chest pain with a confirmed diagnosis of myocardial ischemia/infarction	III	C
Evaluation of chest pain in patients whose non-cardiac etiology is evident	III	C

* Complementation with transesophageal echocardiography increases accuracy and can provide additional information to the transthoracic one; &in the absence of preexisting structural heart disease, prior myocardial infarction, or left bundle branch block.

#### 5.2.2. Stress Echocardiography

The evidence of a new alteration of the segmental contractility at rest or its appearance before the induction of stress (exercise or pharmacological) suggests ischemic etiology.^184^ Stress echocardiography is an independent predictor of cardiovascular death, of additional value to the other methods and can avoid coronary angiography.^175,179^ Its use may be recommended for risk stratification of patients in chest pain units (Table 34), especially when the electrocardiogram does not define the diagnosis and the exercise test is submaximal, non-feasible or inconclusive.^179^ Traditionally, stress echocardiography is performed after 24 hours of chest pain relief in low to moderate risk patients with no evident ischemic changes on the electrocardiogram and normal cardiac enzymes.

**Table 34:** Recommendations of stress echocardiography in acute coronary syndrome

Recommendation	Class of recommendation	Level of evidence
Patients with clinically controlled low risk unstable angina* before deciding the invasive strategy	IIa	A
To assess the functional significance of moderate coronary obstruction at angiography, as long as the result interferes with the procedure	IIa	C
Risk stratification after uncomplicated myocardial infarction	IIa	A
Investigation of patients with suspected microvascular disease& to establish whether segmental change occurs in conjunction with angina and electrocardiographic abnormalities	IIa	C
Strain and strain rate parameters derived from speckle tracking as an adjunct tool to wall motion score index for diagnosis and/or prognosis of acute coronary disease	IIa	B
High risk unstable angina or acute myocardial infarction	III	C

* No recurrence of angina, no signs of heart failure, no abnormalities on the initial/serial electrocardiogram and normal troponin; &typical angina pain with electrocardiogram change or functional test, in the presence of normal coronary angiography

#### 5.2.3. Contrast Echocardiography

This echocardiographic modality allows the immediate and simultaneous access of LV segmental contraction and myocardial perfusion.^179,180^ In patients with acute chest pain and non-diagnostic electrocardiogram, the use of contrast echocardiography increases sensitivity for the diagnosis of ACS (Table 35).180,185 Patients with normal perfusion and myocardial function at rest have good prognosis, while the presence of perfusion defects at rest identifies a subgroup at high risk for ACS.^185^

**Table 35:** Recommendations of contrast echocardiography in acute coronary syndrome

Recommendation	Class of recommendation	Level of evidence
Use of echocardiographic contrast for better definition of the endocardial border and to access left ventricular structure/function when two or more adjacent segments are not visible in the standard resting or stress tests	I	B
Assessment of patients with acute chest pain and non-diagnostic electrocardiogram	IIb	B
Assessment of myocardial perfusion in all types of ACS	III	C
Routine use of echocardiographic contrast in all patients with chest pain and suspected CAD	III	C

ACS: acute coronary syndrome; CAD: coronary artery disease

### 5.3. Chronic Coronary Artery Disease

#### 5.3.1. Transthoracic Echocardiography

TTE, while providing important information on segmental contractility when performed at the time of acute chest pain, is limited in the investigation of patients with chronic coronary disease.^175^ Two situations of indication should be valued: the first one when there is a need for a differential diagnosis of chest pain with non-ischemic causes, such as pericarditis, valvular diseases (such as aortic stenosis) or cardiomyopathies that may occur with chest pain; the second is based on the knowledge of global left ventricular function as a prognostic factor in stable patients with chronic coronary disease (Table 36).^175^

**Table 36:** Recommendations of transthoracic echocardiography in chronic coronary disease

Recommendation	Class of recommendation	Level of evidence
Differential diagnosis of precordial pain	I	B
Initial assessment of left ventricular function as a prognostic indicator, even in patients with no evidence of heart failure	I	B
Assessment of left ventricular function when there is evidence of heart failure or change in clinical status	I	B
Periodic reassessment of stable patients without clinical change	III	C

#### 5.3.2. Stress Echocardiography

Stress echocardiography is a useful investigative method for both suspected patients and those with an established diagnosis of stable coronary disease (Table 37). The method offers good accuracy in the ischemic investigation of patients from moderate to high risk, with a slight predominance of specificity compared to other non-invasive imaging methods, such as myocardial scintigraphy.^179,184,186,187^ The stress-induced modality, whether physical, with treadmill or bicycle, or pharmacological, with dobutamine sensitized with atropine or even with dipyridamole, does not significantly change the diagnostic performance of the test.^179^ In general, in the investigation of stable coronary disease, stress echocardiography should be indicated for those patients with limited exercise performance, either by functional class or by non-interpretable electrocardiogram, such as in the presence of left bundle branch block.^188^ Thus, the method should not be considered as a substitute for the ergometric test. However, if available, it can be used as the first examination in the investigation of selected patients, with intermediate or high pretest probability. Furthermore, in the investigation of the risk of chronic coronary disease, it can be used as a sequential method to others such as coronary tomography, when the calcium score shows levels above 400.^184,186^ Another important clinical situation for indication of stress echocardiography is the preoperative evaluation of patients undergoing intermediate-risk surgery.^179,187,189^ Even in this situation, the method can be used but it should not generally replace the exercise test when this is possible. However, in vascular surgeries, where there are one or more risk factors, the investigation may start from the echocardiographic examination under stress.^184^ Situations in which there is a need for the topographical definition of ischemia, such as those of a functional significance investigation of already known lesions, also induce the image examination under stress, which may be the echocardiography. However, the availability of the method in the region of medical practice and the technical ability and experience of the echocardiography laboratory for individualized application of the levels of recommendation suggested here should be considered.

**Table 37:** Recommendations of stress echocardiography in patients with known or suspected chronic coronary disease

Recommendation	Class of recommendation	Level of evidence
As an initial method in the investigation of chronic coronary disease in patients with intermediate or high pre-test probability	I	B
As an initial method in the investigation of chronic coronary disease in patients with low pretest probability, but unable to perform an exercise test or with electrocardiogram not interpretable	I	B
As a sequential method in the investigation of patients submitted to an ergometric test with intermediate or non-diagnostic result	I	B
As a sequential method in the investigation of patients submitted to coronary tomography with a calcium score (Agatston) > 400	I	B
As a sequential method in the investigation of patients submitted to coronary angiography with identified lesions of uncertain functional significance	I	B
In the preoperative evaluation of non-cardiac vascular surgery in a patient with one or more risk factors for chronic coronary disease	I	B
In the assessment of viability in patients with ventricular dysfunction and chronic coronary disease and eligible for revascularization	I	B
In the preoperative assessment of non-cardiac intermediate risk surgery in a patient with one or more risk factors for chronic coronary disease with functional class < 4 METs or indeterminate	IIa	B
In the sequential investigation of patients with moderate to high pre-test risk with previous testing for ischemia for more than two years	IIa	B
In the assessment of asymptomatic patients after incomplete revascularization	IIa	C
In the evaluation of symptomatic patients after revascularization	IIa	B
Routine reassessment (every five years) in asymptomatic patients after revascularization	IIb	C
As an initial method in the assessment of patients with low pre-test probability and with clinical conditions and interpretable electrocardiogram to perform an ergometric test	III	C
In the preoperative assessment of non-cardiac intermediate-risk surgery in patients with functional class ≥ 4 METS	III	B
Routine ergometric test substitution in patients with physical capacity and interpretable electrocardiogram for the performance of the test	III	C

MET: metabolic equivalent of risk.

#### 5.3.3. Contrast Echocardiography

The use of echocardiographic contrast agents consisting of microbubbles capable of overcoming the pulmonary barrier and remaining intact has become a powerful weapon for adequate visualization of the endocardium of all LV segments.^185,190^ Thus, in the presence of two or more contiguous segments with limited technical quality, the use of any of these agents is indicated (Table 38).^191,192^ The use of contrast agents for myocardial perfusion, although it is part of the same procedure, only with modifications in the acquisition and analysis of the images, remains considered off-label by the US and European health agencies. Guidelines on stable coronary disease already recognize the use of contrast agents to delineate endocardial borders, but they are still not based on myocardial perfusion evaluation.^175^ However, robust evidence supports the use of these agents for assessing myocardial perfusion both in the diagnosis of coronary disease in acute and chronic coronary syndromes,^193^ even showing its superiority over conventional stress echocardiography in predicting cardiovascular events.^194^ The accuracy of contrast agents has been compared with other methods such as myocardial scintigraphy and, in contrast, showed similarity, with greater sensitivity, mainly in the detection of uni-coronary lesions.^195^ However, the use of contrast agents for perfusion analysis is facilitated when stress is performed with dipyridamole; and the low use of dipyridamole in most Brazilian and worldwide echocardiography laboratories^192^ may be a barrier to the implementation of myocardial perfusion analysis.

**Table 38:** Recommendations for the use of echocardiographic contrast agents in chronic coronary disease

Recommendation	Class of recommendation	Level of evidence
Improvement of endocardial border delineation and analysis of global or regional ventricular function when endocardial visibility in two or more segments is limited	I	B
Myocardial perfusion analysis in the diagnosis of chronic coronary disease, both in the assessment of ischemia and viability as an adjunct to the modalities of stress echocardiography	IIa	B
Assessment of the coronary flow reserve in the study of the functional repercussion of already known or viable coronary lesions	IIa	B
Use in the presence or suspected of significant intracardiac shunts	III	B
Routine use of contrast in patients whose image and endocardial edge delineation of the left ventricle are of adequate quality	III	C

Coronary flow reserve can also be assessed by contrast echocardiography under stress. Reduced values of coronary flow reserve are indicative of functional repercussion in lesions that anatomically have dubious expression. In addition, some studies have shown the role of this index in assessing viability and in predicting myocardial functional recovery in patients with stable coronary artery disease.^196^

## 6. Evaluation of Emboligenic Sources and Cardioembolic Diseases

Stroke is the major cause of disability and the second leading cause of death in the world.^197^ Brazil is the Latin American country with the highest mortality rates due to stroke, being the main cause of female death.^198,199^ Although the death rate from stroke has declined in recent decades, the values ​​remain very high.^200^ It is estimated that cardioembolic disease is responsible for 15% to 40% of all causes of ischemic stroke,^201^ whereas indeterminate (cryptogenic) causes account for 30 to 40% of these ischemic neurological events.^202-204^ Other causes of ischemic stroke include large artery atherosclerosis, small vessel occlusion (lacunar), and other etiologies.^205^ In patients who are at risk or who have already had embolic neurologic events, the main role of echocardiography is to identify the presence of an emboligenic source, to determine the probability of such a source being a possible cause of ischemic stroke or systemic embolism and to guide the therapy of these patients. We can classify cardiac diseases as for their emboligenic potential under high and low risk conditions (Table 39). The main causes of ischemic stroke of cardioembolic origin are: atrial fibrillation, associated or not with rheumatic MS (five-fold risk of stroke); left ventricular dysfunction (two to three times greater risk of ischemic stroke than the general population);^206^ acute myocardial infarction (the risk is possibly decreasing by the implementation of early reperfusion therapies);^207^ mechanical valvular prostheses (annual risk of ischemic stroke at 4.0%);^208^ and infectious endocarditis (one in five cases are complicated by ischemic stroke).^209^ Variable rates of annual recurrence of ischemic stroke have been reported in patients with aortic arch atheroma (less than 3 to 12%).^210^ The patent foramen ovale (PFO) can serve as a passageway from a paradoxical embolism of the venous to the arterial circulation. Although patients with an ischemic stroke of indeterminate (cryptogenic) etiology have a higher incidence of FOP than those with known ischemic stroke cause,^211^ a large study reported that the presence of a PFO was not associated with an increased risk of recurrence of ischemic stroke.^212^ Other more rare causes of embolism include papillary fibroelastoma, myxoma, and mitral calcification.

**Table 39:** Classification of cardiac diseases regarding their emboligenic potential

High risk	Low risk
Intracavitary thrombus	Patent foramen ovale
Atrial fibrillation	Interatrial septum aneurysm
Acute myocardial infarctation	Interatrial communication
Dilated cardiomyopathies	Spontaneous contrast
Infectious endocarditis	Lambl excrescences
Valve prostheses	Mitral valvular calcification
Rheumatic mitral stenosis	Aortic valve calcification
Left atrial myxoma	Endocarditis marantica
Papillary fibroelastoma	Mitral valve prolapse
Ulcerated plaques in the aorta	

The cardioembolic etiology of ischemic stroke should be suspected in the presence of severe onset of early neurologic deficit without prodromes, multiple brain lesions in multiple vascular territories, and recurrent ischemic stroke in a short period of time.^213^ Systemic embolization to other organs such as spleen and kidneys at the time of the ischemic stroke increases the suspicion of cardioembolic etiology.^213^ TTE and/or TEE should be recommended in patients with suspected cardiac embolic source, including ischemic stroke and transient ischemic attack (TIA) or systemic embolism. TTE is more suitable for evaluation of embolic sources present in previous cardiac structures, such as the apical thrombus investigation of the left ventricle. In contrast, during the TEE, the transducer is positioned in the esophagus, and the probe is closer to the posterior heart structures. The esophagus is also adjacent to the LA, so TEE corresponds to the gold standard examination for thrombus screening in the left atrial appendix, with sensitivity and specificity approaching 99%. TEE should be recommended as an initial diagnostic tool in the assessment of cardiac embolic source in patients with ischemic stroke, especially in those where the therapeutic decision (anticoagulation or cardioversion) will depend on the echocardiographic findings. TEE should also be recommended when TTE imaging is of poor quality in young patients with ischemic stroke, in patients with ischemic stroke of undetermined etiology, and in those with non-lacunar ischemic stroke. TTE may not be useful when TEE is already programmed for TEE, such as in the evaluation of intracardiac masses, prosthetic heart valves, the thoracic aorta, or to guide percutaneous procedures. TEE should not be recommended when TTE findings are compatible with the embolic cardiac source. Both TTE and TEE should not be recommended in patients whose results will not guide the therapeutic decision. Table 40 lists the main recommendations of TTE and/or TEE in patients with TIA, ischemic stroke or systemic embolism.

**Table 40:** Recommendations of the echocardiography in the evaluation of emboligenic sources and cardioembolic diseases

Recommendation	Class of recommendation	Level of evidence
Suspected cardiac embolic source, including ischemic stroke and TIA or systemic embolism	I	C
Young patient (< 45 years) with TIA or acute ischemic stroke	I	C
Elderly patient with evidence of non-lacunar ischemic stroke	I	C
TIA or cryptogenic ischemic stroke	I	C
TEE as the initial test to facilitate clinical decision-making regarding treatment (anticoagulation or cardioversion)	I	B
Assessment of cardiac emboligenic source when non-cardiac origin has been previously identified	IIb	C
TTE when TEE is already programmed (e.g. in the evaluation of intracardiac masses, prosthetic heart valves, thoracic aorta, or to guide percutaneous procedures)	IIb	C
TEE when TTE findings are diagnostic of cardiac embolic sources	III	C
TTE and/or TEE results do not guide the therapeutic decision	III	C

TIA: transient ischemic attack; TEE: transesophageal echocardiography; TTE: transthoracic echocardiography.

## 7. Atrial Fibrillation

Atrial fibrillation (AF) is the most common sustained cardiac arrhythmia, whose prevalence increases with advancing age.^214,215^ In the United States, it is estimated that the prevalence of atrial fibrillation will double from 5.2 million cases in 2010 to 12.1 million cases in 2030.^216^ In addition to population aging, the increased prevalence of AF can be explained by the comorbidities and associated cardiovascular risks such as hypertension, heart failure, coronary artery disease, valve diseases, obesity and diabetes mellitus.^217^ The risk of developing AF is 1 in 4 individuals from 40 years of age on.^218^ Recent national and international guidelines have reported the classification of AF based on its presentation, duration and spontaneous termination of AF episodes.^214,219,220^ Paroxysmal AF is defined as that which is reversed spontaneously or with medical intervention until seven days after its initiation. Episodes lasting longer than seven days are referred to as persistent AF. Persistent long-term AF represents cases lasting more than one year. Permanent AF corresponds to the cases in which attempts to revert to sinus rhythm will no longer be instituted. Finally, non-valvular AF is defined as AF in the absence of rheumatic MS, mechanical or biological valve or previous mitral valve repair. As part of the initial evaluation, all AF patients should have a TTE to identify structural heart diseases, including valvular heart disease, assess RA and LA size, LV and RV size and function.^184^ TEE is the most sensitive and specific technique for detecting intracavitary thrombi, especially in the left atrial appendage, as a potential source of systemic embolism in AF, and can be used to guide early cardioversion or catheter ablation procedures.^214,220,221^ TEE can also identify features associated with an increased risk of thrombus formation in LA, including reduced flow velocity in the left atrial appendage, spontaneous contrast in LA, and aortic atheroma.^214^ Table 41 lists the main recommendations of TTE and/or TEE in patients with AF. Although the echocardiography provides important information for assessing the likelihood of achieving successful rhythm control after cardioversion, including atrial size, left ventricular systolic function, and severity of valve disease, randomized trials with a larger sample size are still lacking to understand the real prognostic value of imaging techniques in patients with AF.^222^ New echocardiographic techniques such as LA evaluation by strain and 3D echocardiography are promising tools for future clinical practice.^222^

**Table 41:** Recommendations of echocardiography in patients with atrial fibrillation

Recommendation	Class of recommendation	Level of evidence
TTE in the initial assessment of all patients with AF to identify structural heart disease and guide treatment	I	C
TTE in patients with AF lasting ≥ 48 hours to decide early cardioversion with brief heparinization, without previous oral anticoagulation	I	B
TEE in the assessment of patients before ablation or percutaneous occlusion of the left atrial appendage	I	B
Patient with AF requiring emergency cardioversion due to hemodynamic instability	III	C

TTE: transthoracic echocardiography; AF: atrial fibrillation; TEE: transesophageal echocardiography.

## 8. Heart Tumors and Masses

The cardiac masses comprise a broad set of lesions which may be neoplastic and non-neoplastic in nature. As regards incidence, the most frequent causes of cardiac masses are thrombi and vegetation, and rarely are tumors and pseudotumors (intrinsic and extrinsic structures that mimic a cardiac tumor).^223^ Cardiac tumors are extremely rare, with secondary tumors (metastatic neoplasms) being 20 times more frequent than primary tumors.^224,225^ Although the classification of these lesions for benignity or malignancy is an important predictor of prognosis, any cardiac tumor may have substantial hemodynamic or electrical consequences depending on size and location.^226^ Most are detected incidentally during cardiac imaging tests or after complementary evaluation of specific clinical situations, such as after embolic event, suspected endocarditis and the possibility of malignancy involving the heart. Myxomas are the most frequent benign primary tumors in adults, followed by fibroelastomas and, finally, fibromas (much more common in the pediatric population). Primary malignant tumors, however, represent a much smaller portion of primary cardiac neoplasms, with sarcomas and lymphomas being more common. Much more frequent, as mentioned above, are secondary tumors, represented by metastases, which can occur by various forms of dissemination (hematogenous, contiguous, venous and lymphatic), associated mainly to tumors of the breast, lung, esophagus, mediastinum and melanoma. In these cases, the involvement of the pericardium occurs most of the time.^227^

Echocardiography, because of its availability and applicability, is the imaging technique commonly chosen for diagnosis (Table 42). The examination may delineate the multiple cardiac structures and characteristics of a mass, such as location, mobility, morphology, size, insertion site, and potential hemodynamic consequences. It also allows for serial images over time without the need for contrast agents (iodine or gadolinium) or radiation. New techniques, such as the 3D modality, by providing additional anatomical data are capable of increasing the diagnostic accuracy of the method, assisting in the surgical strategy, as well as monitoring immediate and late results of the procedure.^228,229^ The contrasted echocardiographic study represents a very useful tool, offering greater anatomical detail and assisting in the differentiation of the masses through the analysis of its vascularization (hypervascularization is frequently associated with the presence of malignancy).^230^

**Table 42:** Recommendations of the echocardiography in patients with intracardiac masses and tumors

Recommendation	Class of recommendation	Level of evidence
Assessment of individuals with clinical suspicion (signs and symptoms) or patients with conditions predisposing to cardiac tumors	I	C
Carriers of malignant neoplasia with high risk of cardiac involvement	I	C
Evolutionary follow-up after surgical removal of cardiac tumors with high potential for recurrence (such as myxomas)	I	C
TTE for complementary anatomical and functional assessment in cases in which TTE was not definitive	I	C
TEE for complementary intraoperative assessment	I	C
3D echocardiography to search for additional anatomical information not seen in 2D TTE	I	C
Use of echocardiographic contrast for differential diagnosis and vascularization analysis	IIa	B
Patients with direct relatives with family history of myxoma	IIa	B
Patients whose results of the examination findings will not imply in therapeutic decision	III	C

TEE: transesophageal echocardiography; TTE: transthoracic echocardiography; 3D: three dimensional; 2D: two dimensional.

## 9. Pericardial Diseases

Echocardiography should be indicated in the suspicion of pericardial affections, including (but not only) pericardial effusion, pericardial mass, constrictive pericarditis, effusive-constrictive pericarditis, patients after cardiac surgery and suspicion of cardiac tamponade (Table 43).^231,232^ It contributes decisively to the semiquantitative evaluation of the pericardial effusion and its hemodynamic repercussion (depending on the volume and the velocity of the collected fluid), as well as exploring the underlying etiology, whether primary (e.g. pericarditis, chylothorax) or secondary (e.g. bleeding, metastasis, myxedema, hydropericardium). The method provides information about the nature of the fluid, suggesting the presence of fibrin, clot, tumor, air, and calcium. The size of the effusion may be classified by the diastolic measurement of the echo-free pericardial space , as of small (< 10 mm), moderate (10 to 20 mm) and large (> 20 mm).^233^ Findings indicative of cardiac compression may precede the clinical manifestations of the tamponade and configure an emergency situation. In this context, pericardial puncture guided by echocardiography may alleviate hemodynamic impairment and save lives.^233^ Such procedure can be performed safely in centers with experience, avoiding radiation associated with fluoroscopy and/or cost of surgery, which makes pericardiocentesis guided by echocardiography the procedure of choice.^234^ Individuals with chronic or recurrent pericardial effusion, not responsive to the proposed clinical treatment, may be referred for elective pericardial drainage after serial evaluation. The spectrum of echocardiography utilization in pericardial disease also includes congenital defects, trauma, neoplasia, cysts, CT after radiotherapy and the differential diagnosis between constrictive pericarditis and restrictive cardiomyopathy. In this differentiation, findings compatible with constriction are: exacerbated decrease (> 25%) in the E-wave velocity of the mitral flow in the first beat after inspiration, normal tissue Doppler mitral annular velocity (e’ > 7 cm/s) and annulus paradoxus (e’ septal > e’ lateral).^233^

**Table 43:** Recommendations of the echocardiography in pericardial diseases

Recommendation	Class of recommendation	Level of evidence
Clinical suspicion of pericardial effusion	I	C
Serial studies for evaluation of recurrent stroke	I	C
Assessment after radiotherapy (five years in patients at high risk for cardiotoxicity and ten years in others)	I	C
Suspicion of constrictive pericarditis, early detection of constriction or differential diagnosis with restriction	I	B
Suspected cardiac tamponade (chest trauma, cardiac surgery, iatrogenic perforation in cardiac catheterization or electrophysiological study, rupture of the ventricular wall after myocardial infarction and aortic dissection)	I	C
Suspected pericardial cyst, pericardial mass or pericardial agenesis	I	C
Monitoring of pericardiocentesis	I	B
Serial studies to assess the effect of treatment on stroke	IIa	C
Routine examination for small effusions in hemodynamically stable patients	III	C
Investigation of pericardial thickening without repercussion	III	C

## 10. Systemic Diseases

### 10.1. Introduction

The indication of echocardiography in systemic diseases depends on the prevalence of associated heart disease, the characteristics peculiar to cardiac involvement in each situation and the clinical suspicion of cardiac involvement.^235^ For example, examination is mandatory in individuals with systemic diseases potentially causing restrictive cardiomyopathy that show signs and symptoms of heart failure in clinical evolution. Some systemic diseases for which the indication of the examination should be considered are as follows.

### 10.2. Chronic Renal Failure

Morphophysiological changes in LV (such as hypertrophy, dilatation, systolic and diastolic dysfunction) are common in patients with end-stage renal disease and predict a worse prognosis.^236-238^ International guidelines recommend TTE for all dialysis patients one to three months after the initiation of renal replacement therapy and at three-year intervals thereafter, despite the symptoms.^239^

### 10.3. Amyloidosis

It is a common cause of restrictive cardiomyopathy and may be familial (transthyretin) or nonfamiliar (primary or light chain). Cardiac involvement due to amyloid deposition may lead to some suggestive echocardiographic findings: thickening and increased echogenicity (“granular” appearance) of the LV walls (especially in the absence of arterial hypertension), biatrial dilatation, thickening of the valves and interatrial septum, diastolic dysfunction (grade II and III), small pericardial effusion, prominent decrease in longitudinal strain in the basal and mid LV segments (“sparing” the apical segments), and later systolic dysfunction with LVEF.^240^

### 10.4. Sarcoidosis

It is important to investigate the presence of cardiac involvement in sarcoidosis (granulomatous disease of unknown origin), as this is a potentially fatal condition. Among the various echocardiographic findings that may be found, we have: dilated cardiomyopathy, restrictive cardiomyopathy, segmental contractility alterations that do not obey the classic coronary territorial distribution, basal septum akinesia, inferolateral aneurysm and abnormal thickness of the septum (thickening or thinning).^241^

### 10.5. Neoplasias

The echocardiography can detect silent pericardial metastases in some types of neoplasia (such as breast and lung) and monitor the cardiotoxic effect of chemotherapeutic agents.^242^

### 10.6. Autoimmune Diseases

The test may diagnose lupus-associated cardiac manifestations, such as pericardial effusion and sterile vegetations, systemic sclerosis, such as pulmonary hypertension, or rheumatoid arthritis, such as valve abnormalities.^235^

## 11. Diseases of The Aorta, Pulmonary Artery and Veins

### 11.1. Aorta

The evaluation of the aorta is routine in the TTE, since it allows to examine some of its segments, mainly the aortic root and the proximal portion of the ascending aorta, affected in numerous affections. The root of the aorta is formed by the aortic ring, the sinuses of Valsalva and the synotubular junction. The descending aorta and the proximal abdominal aorta can be evaluated at the suprasternal and subxiphoid sections, respectively.^154,243^ However, the TTE should be considered a screening test, with limitations, since it does not allow the analysis of all segments of the aorta, such as the aortic arch and the distal descending. In this case, it is necessary to use other imaging methods such as TEE, computed tomography or magnetic resonance imaging.^154,243^ The TEE allows the realization of excellent resolution images due to the proximity of the esophagus to the thoracic aorta. Despite the multiplanar sections offered, a small segment of the ascending aortic junction with the aortic arch is not visualized due to the interposition of the trachea.^154^ The precise diagnosis of acute aortic syndromes, such as aortic dissection, intramural hematoma, penetrating ulcer and rupture of aortic aneurysms, is fundamental in the therapeutic strategy to be adopted. In unstable patients with suspected acute aortic syndrome, the imaging modality chosen will depend on local availability and expertise. In general, TTE is performed as an initial investigation (class I), complemented with TEE and/or tomography (both class I). The TEE shows good accuracy, mainly in the exclusion of artifacts caused by reverberations of the anterior wall of the LA and the pulmonary artery.^244^ Depending on the clinical suspicion, the diagnostic investigation should proceed with two or more imaging examinations due to the possibility of false negatives.^245^

### 11.2. Pulmonary Artery

The trunk of the pulmonary artery and the initial portion of the pulmonary branches can be assessed to TTE. TEE is more accurate, allowing a greater examination of the pulmonary artery and its branches, which allows a better appreciation of thrombi in the proximal territory of the pulmonary artery. Dilations of these vessels can also be diagnosed. Pulmonary artery dilatations are uncommon lesions and may be associated with different etiologies, such as congenital heart diseases, systemic vasculitis, collagenosis, infections and traumas.

### 11.3. Veins

Anomalies of the superior and inferior vena cava can be diagnosed by TTE and/or TEE. The presence of thrombi in these pathways and the extension of tumors into the right cavities can be evaluated. TEE is particularly useful for the identification of thrombus or vegetation in the superior vena cava in patients with long-stay catheters and in cases of pulmonary vein stenosis after AF or atrial flutter ablation procedures. The persistence of the left superior vena cava should be suspected in the presence of dilated coronary sinus and the diagnosis can be made using intravenous injection of agitated saline solution, which will first contrast the coronary sinus and then the right cavities. In this case, it is important to emphasize the need to exclude anomalous drainage of the left pulmonary vein via the vertical vein.^246^

## 12. Intraoperative Echocardiography in Cardiac and Non-Cardiac Surgeries

### 12.1. Introduction

Intraoperative echocardiography is a technique for monitoring cardiac and non-cardiac surgeries that allows a rapid and real-time assessment of anatomic and functional cardiac features (global and segmental function, valvular function, volume and vascular resistance), aortic and phenomena with embolic potential.^247,248^ In non-cardiac surgeries, clinical information obtained by intraoperative echocardiography is often complementary to data provided by other hemodynamic monitoring devices (e.g., central venous catheter, pulmonary artery catheter, or arterial line).^247,249^ In the case of cardiovascular surgeries, the intraoperative echocardiography can also contribute with real-time dynamic information and images of the cardiac structures to plan, guide and evaluate the immediate result of the surgical intervention.^250^

### 12.2. Modalities of Intraoperative Echocardiography

- Transesophageal: most widely used in open, minimally invasive or percutaneous cardiac surgeries, as well as in non-cardiac surgeries. It has the advantage of not entering the sterile field and of not disturbing the surgical procedure, allowing continuous monitoring. It is a relatively safe modality when performed by properly trained professionals. The contraindications are the same as for the conventional TEE. In young children, the use of intraoperative TEE should be considered on a case-by-case basis, based on the unique risks of these patients (e.g., bronchial obstruction).^248^
- Epicardial or epiaortic: are an alternative for monitoring open heart surgeries in which there is absolute or relative contraindication of manipulation of the esophagus, or blood dyscrasia. In these embodiments, the linear or sectoral transducer is wrapped in a sterile cap and applied directly over the heart or aorta. The epiaortic technique is a very important tool in patients with advanced atheromatous disease, since it allows the choice of a suitable site for cannulation and aortic clamping.^249^
- Transthoracic: may be considered as a monitoring alternative for percutaneous or non-cardiac procedures that are performed with superficial sedation, or in cases in which the patient has absolute or relative contraindication of esophageal manipulation. In this modality, the examination can be performed serially during the procedure, or at specific times as needed (e.g., in cases of hemodynamic instability, to guide endomyocardial biopsy).
- Intracavitary: little used, more restricted to percutaneous procedures.

### 12.3. Recommendations in Cardiac and Thoracic Aorta Surgery

The main objectives of the use of intraoperative echocardiography in cardiac and thoracic aorta surgeries are: to confirm and refine the preoperative diagnosis; to detect new or unsuspected morphophysiological alteration; adjusting the surgical or anesthetic plane according to the findings; to guide the positioning of cannulae or devices; to evaluate the presence of air, masses and thrombi in cardiac cavities and their embolic potential; to evaluate segmental and global left ventricular function and cavitary pressures; and to evaluate the immediate outcome of the intervention.^244,245,249^ The main recommendations in this scenario are in the Table 44.

**Table 44:** Recommendations of the intraoperative echocardiography in cardiac and thoracic aorta surgeries

Recommendation	Class of recommendation	Level of evidence
Adults submitted to cardiac or thoracic aorta open-chest surgery (e.g., valve plasty replacement, structural, mass resection, correction of aortic dissection or aortic aneurysm)	I	B
Adults undergoing minimally invasive cardiac surgery	I	B
In young children undergoing open or minimally invasive cardiac surgery, the indication should be considered case by case according to the risks of intraoperative echocardiography in the pediatric population (e.g., bronchial obstruction)	I	B
Placement and adjustment of ventricular assist devices	I	B
Myocardial revascularization surgery when there is left ventricular systolic dysfunction	IIa	B
In case there is a trained professional available, three-dimensional echocardiography should always be used for valvular (aortic and mitral regurgitation), structural and mass resection procedures	I	B

### 12.4. Recommendations in Non-Cardiac Surgeries

The main objectives of intraoperative echocardiography in non-cardiac surgeries are: to assess volume status and fluid response; to estimate vascular resistance and cavitary pressures; to evaluate function of the ventricles (global and segmental) and valves; and to investigate special issues according to the clinical status and the type of intervention.^244,245,249^ The main recommendations in this scenario are found in Table 45.

**Table 45:** Recommendations of the intraoperative echocardiography in non-cardiac surgeries

Recommendation	Class of recommendation	Level of evidence
Intraoperative echocardiography may be indicated for the monitoring of non-cardiac surgeries if the patient is suffering from severe cardiovascular disease and/or the planned procedure may result in severe hemodynamic, myocardial, pulmonary or neurological impairment	I	B
Large vascular surgery (usually in open abdominal aortic repair surgeries)	I	B
Liver transplantation	IIa	B
Pulmonary transplantation	IIa	B
Renal tumor resection	IIb	B
Surgery of trauma	IIb	B
Neurosurgery	IIa	B
Orthopedic Surgeries	IIb	B
Laparoscopic surgeries	IIb	B

## 13. Echocardiography in Percutaneous Interventions

### 13.1. Introduction

In general, cardiac interventions through cardiac catheterization have developed with the support of the echocardiographic image, especially by the transesophageal route. Its use in daily practice is increasingly frequent, in view of the development of techniques and technologies for the treatment of diseases previously only corrected by the conventional surgical procedure. In addition, there was remarkable progress in the diagnostic capacity of echocardiography, mainly due to the improvement of the image quality and the advent of the 3D image, obtained by TTE and TEE. The examination has practically no contraindication and allows the early identification of potential complications. There is no randomized study for non-use of echocardiography during procedures, and some are limited to the use of thoracic, esophageal or intracardiac modalities. Therefore, in relation to interventional procedures, indication of echocardiography use is in principle class I, level of evidence C, a fact already recognized in the literature.^234^ In some examinations, it becomes necessary as well as essential, since the X-ray (XR) image is insufficient to perform the procedure, and sometimes even expendable. The role of echocardiography in major interventional procedures follows below.

### 13.2. Follow-up of Interventions in Congenital Heart Diseases

- Atrial septostomy: also known as Rashkind procedure. The septostomy is performed by balloon in a population in which the TTE allows an optimal visualization of the atrial septum and of the catheter, guiding in an appropriate way and evaluating the immediate result and eventual complications. The use of TEE is usually unnecessary, and also impracticable, due to the lack of adequate probes. In this case, even the XR scoping can be discarded.
- Procedures for occlusion of atrial septal defects: there are devices for occlusion of defects of the atrial septal defect, ostium secundum, as well as patent foramen ovale. The examination can be performed in both, mainly TEE or intracardiac echocardiography, where it is possible to assist in the choice of the device, its positioning, immediate result and rapid identification of complications. TTE may be preferable to TEE in selected patients.^251^
- Procedures for occlusion of ventricular septal defects: TEE should be performed to better understand the anatomical aspects, the procedure itself and possible complications. Acquired ventricular septum defects, such as those after trauma or after acute infarction, may also be treated with the aid of echocardiography, presenting the same value in the procedure.
- Procedures for occlusion of ductus arteriosus persistence: in this situation, echocardiography is disregarded, since catheterization during the procedure is usually sufficient for success.

### 13.3. Electrophysiology Procedures

The echocardiography mainly supports the procedure of puncture of the atrial septum, usually through TEE, or alternatively by intracardiac echocardiography. In the procedure of ablation or implantation of pacemaker the echocardiography is dispensable, since the electrical mapping provides the necessary information. In the presence of complications, such as perforation of a chamber with pericardial effusion and tamponade, the echocardiography is normally requested.

### 13.4. Alcoholic Ablation in Hypertrophic Cardiomyopathy

One of the treatments for symptomatic patients consists of alcohol ablation of the segment where there is a greater degree of hypertrophy and related to intraventricular obstruction in its exit pathway. TTE is more used, and there is no restriction on the use of TEE. Prior to alcohol infusion, the septal artery is catheterized and a solution is infused. At this time, the echocardiography should assess whether the contrast-enhanced myocardial segment corresponds to the portion of the myocardium associated with the obstruction and if it does not occur in all transmurality of the segment, which is undesirable. The Doppler study estimates the gradient of obstruction and also the degree of mitral regurgitation. After alcoholic ablation, we repeat the measurement of gradients and mitral regurgitation, whose falls indicate successful treatment, and possible complications are investigated.

### 13.5. Left Atrial Appendage Occlusion

An alternative, when it is impossible to carry out adequate anticoagulation in patients at high risk for atrial arrhythmia embolism, is occlusion of the left atrial appendage. TEE is mandatory in this treatment the, as it not only helps the transseptal puncture, but also allows adequate appenddage measurements, which select the dimensions of the occluder device. Still in the room, the TEE guides the procedure, confers its result and makes it possible to diagnose complications.

### 13.6. Treatment of Heart Valves

- Percutaneous treatment of mitral regurgitation: among the several treatments proposed, the only one that is commercially available is Mitraclip®. In this treatment there is a mimicry of the Alfieri surgery, where there is the formation of a double mitral orifice. To do so, a metallic clip is inserted through the vein, which advances to the LA after puncture of the atrial septum and is positioned so as to reduce severe mitral regurgitation. In this case, no step is performed without the TEE, considering that the 3D image provides better understanding of the procedure.
- Balloon catheter mitral valvotomy: the use of balloons for the treatment of severe rheumatic mitral stenosis is a safe and efficient alternative. The echocardiography prior to the procedure provides information that can predict the chance of success.^252,253^ In the intervention, the TEE is preferable, and the morphological aspects of the mitral valve should be reviewed. During the inflation of the balloon (or balloons), the echocardiography allows to detect proper positioning. Immediately after the procedure, measures are performed with the objective of evaluating the success of the procedure and the complications that may have occurred.
- Balloon catheter aortic valvotomy: the use of this therapy is currently safeguarded as the last alternative for the treatment of aortic stenosis or as a bridge to compensate for the clinical condition and subsequent implantation of a prosthesis via cardiac catheterization or surgery. The echocardiography can be used to evaluate immediate results and complications.
- Transcatheter implant of aortic valve prosthesis: the indication of the percutaneous implantation of aortic valve prosthesis is conditioned to clinical and morphological aspects of the aortic valve. It is necessary to diagnose severe aortic stenosis prior to the procedure, as well as the analysis of the aortic complex, which selects the size of the most appropriate device for the procedure, directly related to its success. The preference is for the use of TEE, especially with 3D image. During the procedure, it is necessary to review the severity of valve stenosis by measuring the gradients and estimating the effective flow orifice. Still, measurement of the aortic complex and especially of the area/perimeter of the aortic ring, performed only through the 3D echocardiography is necessary. The value obtained is optimally related to tomography measurements that are usually used to select the device.^254^ Whether femoral or transapical, monitoring of the arrival of the prosthesis to the aortic valve is performed, as well as the aid of the ideal positioning, prior to its opening. After implantation, the TEE should provide data on adequate prosthesis expansion, presence and degree of prosthetic and/or paraprosthetic regurgitation. Complications of the procedure are part of the echocardiography investigation.

### 13.7. Treatment of Prosthetic Dysfunction

- Valve in valve: The term “valve in valve” means the implant of a prosthesis via a catheter over a dysfunctioning bioprosthesis. It can be made in prostheses in aortic and mitral position, the latter only by transapical way. The monitoring during the process of arrival of the prosthesis, its implant and the identification of complications must be done by the TEE.
- Occlusion of paraprosthetic regurgitation orifices: one of the possibilities of regurgitation in a valve prosthesis is the presence of paraprosthetic orifices. TEE, especially 3D, should be used to identify these orifices, locate them accurately, and measure their area and diameter to select the most appropriate occlusion device. In the procedure, the echocardiographic image helps to visualize the passage of the guiding catheters through the paraprosthetic site, to open the device and to measure the success of the treatment.

## Figures and Tables

**Table 22:** Recommendations of transthoracic echocardiography in patients with bicuspid aortic valve and ascending aorta dilatation

Recommendation	Class of recommendation	Level of evidence
Assessment of aortic root and ascending aorta diameter in patients with bicuspid aortic valve	I	B
Annual reassessment of size and morphology of the aortic root and ascending aorta in a patient with a bicuspid aortic valve and aortic diameter between 4.0 and 4.5 cm, if the size remained stable within the first 6-month interval after the first exam	I	B
Six-monthly reassessment of the aortic root and ascending aorta size and morphology in a patient with a bicuspid aortic valve and one of the following criteria: aortic diameter > 4.5 cm; rapid increase in aortic diameter (> 0.3 cm); family history of aortic disease in a first degree relative; or if it is the first examination to detect aortic dilatation	I	B

**Table 23:** Recommendations of stress echocardiography in aortic regurgitation^153^

Recommendation	Class of recommendation	Level of evidence
Stress echocardiography in asymptomatic or marked AR with doubtful symptoms to evaluate exercise-induced symptoms and functional capacity	IIa	B
Stress echocardiography in moderate AR with evident or doubtful symptoms to confirm and exclude other causes	IIa	B
Echocardiography under stress with exercise or with dobutamine when there is a discrepancy between the severity of AR to the transthoracic echocardiography and clinical symptoms, to better quantify the AR	III	C

AR: aortic regurgitation
